# Integrative Single‐Cell Analysis Reveals Iron Overload‐Induced Senescence and Metabolic Reprogramming in Ovarian Endometriosis‐Associated Infertility

**DOI:** 10.1002/advs.202417528

**Published:** 2025-07-22

**Authors:** Yangshuo Li, Wei Zhou, Jie Ding, Di Song, Wen Cheng, Jin Yu, Shuai Sun, Shanshan Mei, Xiaolan Liang, Qianqian Zhao, Yanping Kuang, Mingqing Li, Zhexin Ni, Chaoqin Yu, Yue Gao

**Affiliations:** ^1^ Department of Traditional Chinese Gynecology the First Affiliated Hospital of Naval Military Medical University Shanghai 200433 China; ^2^ Beijing Institute of Radiation Medicine Beijing 100850 China; ^3^ Department of Assisted Reproduction the First Affiliated Hospital of Naval Medical University Shanghai 200433 China; ^4^ Department of Gynecology and Obstetrics The First Affiliated Hospital of Zhejiang Chinese Medical University (Zhejiang Provincial Hospital of Chinese Medicine) Hangzhou Zhejiang Province 310006 China; ^5^ Department of Assisted Reproduction Shanghai Ninth People's Hospital Shanghai Jiao Tong University School of Medicine Shanghai 200011 China; ^6^ Department of Reproductive Immunology The International Peace Maternity and Child Health Hospital School of Medicine Shanghai Jiao Tong University Shanghai 200030 China

**Keywords:** endometriosis, infertility, iron, multi‐omics, senescence

## Abstract

Endometriosis, particularly ovarian endometriosis (OE), is a major cause of infertility, often associated with reduced oocyte quality and impaired ovarian function. Iron overload plays a key role in OE progression. This study investigates the effects of iron overload on follicular function in OE‐associated infertility (OEI). A single‐cell atlas of pre‐ovulatory follicular fluid from OEI patients reveals dynamic changes in iron metabolism and iron‐induced senescence phenotypes. Spatial transcriptomics using Stereo‐seq in iron‐overloaded mouse ovaries further identifies localized senescence features. Additional analysis of aging human ovaries highlights conserved patterns of iron dysregulation. These findings provide mechanistic insight into iron overload‐related ovarian pathology and suggest potential therapeutic targets for improving oocyte quality in OEI.

## Graphical abstract

Iron overload in ovarian endometriosis (OE) disrupts follicular function and contributes to infertility. This study maps iron‐induced cellular changes using single‐cell and spatial transcriptomics, revealing senescence phenotypes in OE patients and mouse models. Similar patterns are observed in aging ovaries, highlighting iron metabolism as a potential therapeutic target to improve oocyte quality in OE‐associated infertility.

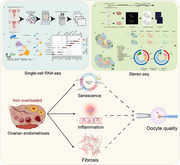

## 1 Introduction

Endometriosis is a prevalent gynecological disorder characterized by endometrial‐like tissue outside the uterus, affecting ≈10% of women of reproductive age.^[^ ^1^, ^2^, ^3^ ^]^ Beyond causing chronic pelvic pain, dysmenorrhea, and dyspareunia, endometriosis is a significant contributor to infertility.^[^ ^1^, ^2^, ^3^ ^]^ Although the general prevalence is 6%–10%, it affects 35%–50% of infertile women.^[^ ^1^, ^4^ ^]^ Ovarian endometriosis (OE), or endometrioma, accounts for 17%–44% of endometriosis cases.^[^ ^5^ ^]^ Embryos fertilized from oocytes of OE‐associated infertility (OEI) patients exhibit lower implantation rates than those from other endometriosis type.^[^ ^6^ ^]^ Morphological changes in oocytes from OE‐affected ovaries, such as cortical granule loss and zona pellucida hardening, further impair fertilization, embryo development, and implantation.^[^ ^7^ ^]^ Despite similar live birth and clinical pregnancy rates in OEI patients undergoing In‐Vitro Fertilization/Intra‐Cytoplasmic Sperm Injection (IVF/ICSI) compared to women without endometriosis, they face higher cycle cancellation rates and retrieve fewer oocytes.^[^ ^5^ ^]^ While assisted reproductive technologies (ART) can help OEI patients select superior embryos, but the damage to oocyte quality and the ovarian microenvironment caused by OE, coupled with reduced ovarian reserve, remains a critical concern.^[^ ^8^, ^9^ ^]^

Emerging evidence links iron overload, may induced by retrograde menstruation, in endometriotic lesions to disease progression, and elevated iron levels also find in the follicular fluid of OEI patients.^[^ ^10^, ^11^, ^12^ ^]^ Excessive iron deposition in OE‐affected ovaries leads to follicular dysfunction and reduced fertility.^[^ ^10^, ^13^ ^]^ Our previous studies identified excess free iron in the follicular fluid of OEI patients as a key factor in infertility.^[^ ^11^, ^12^ ^]^ However, the epigenetic dynamics of different cell types in the follicular fluid of OEI patients under iron‐overloaded conditions remain poorly understood.

Understanding pre‐ovulatory follicle events under iron overload is challenging due to the complex nature of follicular development and the ethical and technical constraints of studying human ovarian tissue.^[^ ^14^ ^]^ Follicular fluid from ART patients, which contains various cells, metabolites, and cytokines, offers a valuable alternative,^[^ ^15^, ^16^, ^17^ ^]^ though ethical challenges persist in obtaining pre‐ovulatory oocytes for research. Investigating granulosa cells, which support oocytes, provides a more feasible approach, as their abnormalities reflect disruptions in oocyte development.^[^ ^18^ ^]^ Although these samples do not capture the full temporal dynamics of in vivo follicular development under iron‐overloaded conditions, their diverse cellular composition helps elucidate the aberrant cell communication and coordination under iron overload.

Recent advances in single‐cell RNA sequencing (scRNA‐seq) technology have enabled exploration of cellular complexity and dynamic interactions in various disease models.^[^ ^19^ ^]^ For instance, single‐cell studies have revealed distinct cell compositions and transcriptional reprogramming in endometriotic lesions, promoting immune tolerance and angiogenesis.^[^ ^20^, ^21^ ^]^ Additionally, spatially enhanced resolution omics sequencing (Stereo‐seq), a form of spatial transcriptomics, provides high‐resolution transcriptomic analysis at the single‐cell level, overcoming scRNA‐seq's limitations in capturing spatial information. For example, high‐resolution spatiotemporal transcriptomics have mapped spatial changes in cell types and specific molecular processes in pre‐ovulatory mouse ovaries.^[^ ^22^ ^]^

In this study, we investigated how iron overload in the follicular microenvironment contributes to oocyte damage in OEI patients through multi‐level validation. First, our retrospective cohort analysis and Smart‐seq2 sequencing identified impaired oocyte maturation as the primary determinant of OEI, building upon bulk RNA‐seq evidence of iron dysregulation. To dissect the cellular mechanisms, we constructed the first single‐cell of pre‐ovulatory iron‐overloaded follicular fluid using scRNA sequencing, systematically mapping iron metabolism dynamics across cell types and revealing iron accumulation‐induced senescence phenotypes. This analysis further delineated iron‐sensitive differentiation trajectories of cumulus cells and uncovered macrophage M1/M2 polarization states linked to iron metabolism. We employed an iron‐overloaded mouse model with Stereo‐seq spatial transcriptomics, which confirmed conserved senescence signatures. Crucially, we extended these findings to human ovarian aging by reanalyzing public scRNA‐seq datasets, demonstrating shared molecular pathways of iron imbalance across pathological and physiological contexts. From clinical observation to cellular mechanism and in vivo validation, our work elucidates how iron overload disrupts follicular homeostasis, while providing actionable targets for therapeutic interventions aimed at improving oocyte quality.

## 2 Results

### 2.1 IVF/ICSI Cohort Analysis

To explore the impact of OE on IVF/ICSI outcomes, particularly its effects on oocyte quality and quantity, we conducted a retrospective analysis of 4,087 patients: 385 with OEI, 2,698 tubal factor infertility, and 1,004 with male factor infertility. To control for confounding variables, propensity score matching^[^ ^23^ ^]^ was utilized (see methods), resulting in 308 patients per group for further analysis. (Table S1, Supporting Information).

**Figure 1:**
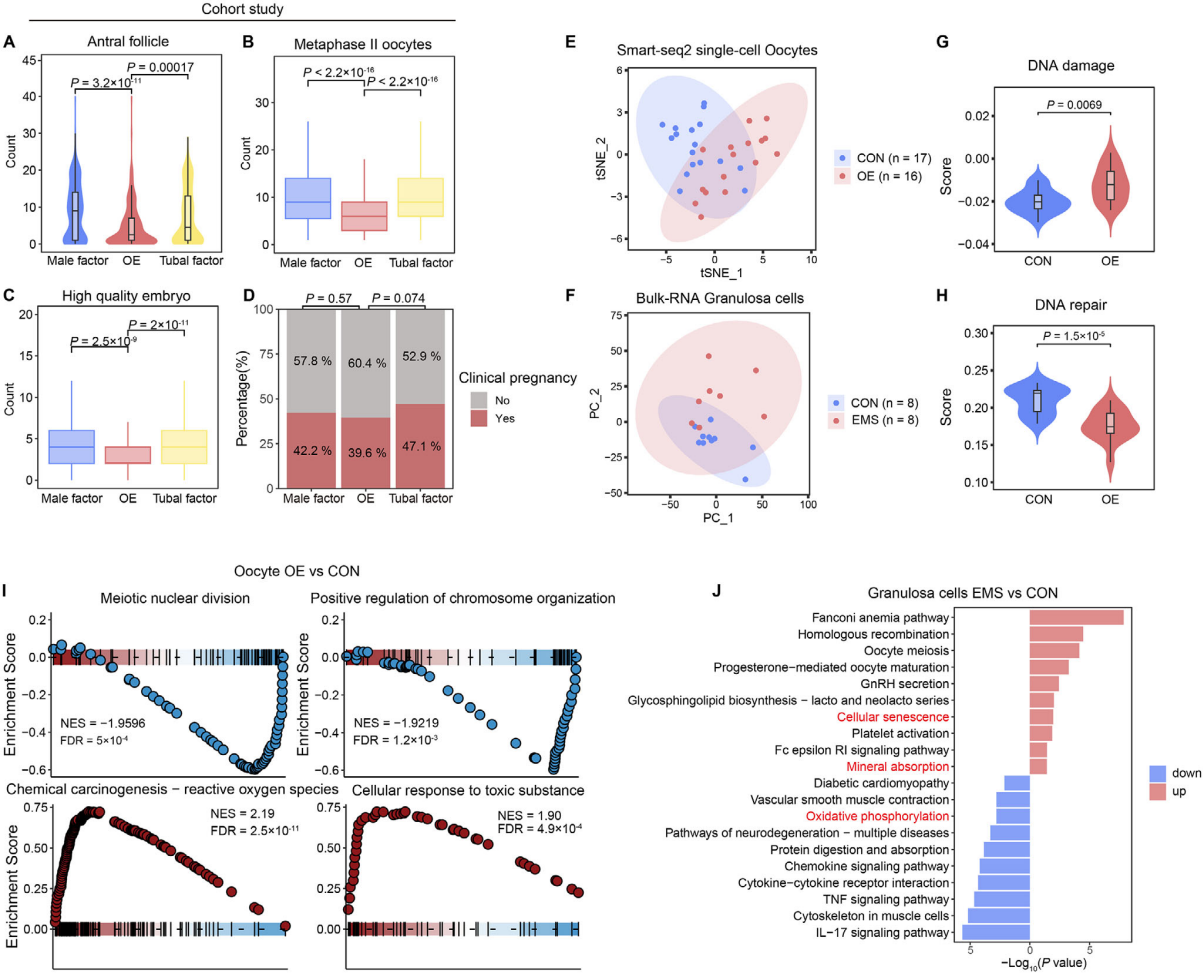
Aberrant follicular development and transcriptional alterations in oocytes and granulosa cells from OEI patients. A–D) Retrospective analysis of 924 IVF/ICSI cycles, categorized into three groups: male factor infertility, ovarian endometriosis (OE), and tubal factor infertility, with 308 patients in each group. (A) Baseline antral follicle count in the three groups prior to IVF/ICSI treatment. Number of mature oocytes (B) and high‐quality embryos (C) retrieved during the treatment. (D) Clinical pregnancy rates post‐IVF/ICSI treatment in each group. Statistical significance for (A‐C) using two‐sided Wilcoxon rank‐sum test and for (D) using Fisher's exact test. E) Distribution of oocytes from the OE group (n = 17) and healthy donors (n = 16, CON) on a *t*‐distributed stochastic neighbor embedding (t‐SNE) plot using Smart‐seq2 single‐cell RNA sequencing. F) Principal component analysis (PCA) of granulosa cells from endometriosis‐associated infertility (EMS, n = 8) and infertility due to other factors (CON, n = 8), based on bulk RNA sequencing data. G,H) Gene set scoring of DNA damage (G) and repair (H) pathways in oocytes from the OE and CON groups. Two‐sided Wilcoxon rank‐sum test. I) Gene set enrichment analysis (GSEA) of oocytes from the OE and CON groups on significant differential pathways. Each point represents a gene within the pathway, with its vertical position indicating its contribution to pathway enrichment. NES > 0 indicates upregulation in the OE group; NES < 0 indicates downregulation. NES, normalized enrichment score; FDR, false discovery rate. () Kyoto Encyclopedia of Genes and Genomes (KEGG) enrichment analysis of granulosa cells from EMS and CON groups. Red (blue) indicates pathways enriched with upregulated (downregulated) genes in the EMS group.

Our findings indicate that the OE group had significantly fewer antral follicles and mature oocytes compared to the other groups (**Figure** 1A,B and Tables S1 and S2, Supporting Information). Despite the critical role of sperm and oocyte quality in embryonic development, the OE group (normal male sperm) produced fewer high‐quality embryos than the male and tubal factor infertility groups (Figure 1C and Table S2, Supporting Information), implying that diminished oocyte quality in OEI patients compromises reproductive potential. Notably, there was no significant difference in live birth rates between the OE and other groups (Figure 1D), consistent with previous reports.^[^ ^5^ ^]^ However, the rates of congenital and neonatal defects were significantly higher in the OE group (Table S3, Supporting Information). In conclusion, our IVF/ICSI cohort analysis demonstrates that OE is linked to impaired oocyte development, potentially affecting ART success rates.

### 2.2 Transcriptome Sequencing Analysis of Follicles from OEI Patients

To investigate potential abnormalities in OEI patient follicles, we integrated public Smart‐seq2 datasets of metaphase II (MII) oocytes from OEI patients^[^ ^24^ ^]^ with our bulk RNA sequencing (RNA‐seq) data of follicular fluid. We excluded data confounded by patient age and BMI to ensure the validity of the public dataset (Figure S1A, Supporting Information) and verified its reliability using known oocyte marker genes such as *ZP1*, *DDX4*, and *BMP15* ^[^ ^25^ ^]^ (Figure S1B, Supporting Information). Our analysis revealed transcriptional heterogeneity in both oocytes and granulosa cells from the OE group compared to the control (CON) group (Figure 1E,F, Figure S1C,D and Tables S4 and S5, Supporting Information). Given the heightened DNA synthesis and replication activity in MII oocytes,^[^ ^26^ ^]^ we evaluated DNA repair and damage gene signatures in OEI patients (Table S6, Supporting Information). We found an elevated DNA damage signature and reduced DNA repair signature in the OE group (Figure 1G,H). Further analysis indicated suppression of biological processes related to “Meiotic nuclear division” and “Positive regulation of chromosome organization” in OE oocytes, with upregulation of processes involving “Reactive oxygen species (ROS)” and “Cellular response to toxic substances” (Figure 1I and Table S7, Supporting Information). Notably, genes associated with oxidative damage, such as *SLC25A5*, *APOE*, and the *PRDX* family, were upregulated in OE oocytes, while genes crucial for successful fertilization, such as *WEE2* ^[^ ^27^ ^]^ and *SIN3A*,^[^ ^28^ ^]^ were downregulated (Figure S1C–F, Supporting Information). These findings suggest that infertility in OEI patients may be linked to abnormal transcriptional regulation in oocytes, particularly concerning oxidative stress, DNA damage, and repair dysfunction during meiosis.

Consistent with previous reports,^[^ ^11^, ^29^ ^]^ we observed upregulation of pathways associated with cellular senescence and metal ion absorption related to iron overload, while oxidative phosphorylation pathways were downregulated in granulosa cells from OEI patients (Figure 1J and Table S8, Supporting Information). Notably, myeloid and lymphoid cell marker were detected in the follicular fluid transcriptome, indicating the presence of immune cells (Figure S1G, Supporting Information). This suggests that traditional RNA‐seq may not fully capture the transcriptional regulation and molecular interactions among different cell types within the follicular fluid. Therefore, we employed scRNA‐seq to further characterize the cellular heterogeneity and pathological mechanisms underlying pre‐ovulatory follicular responses in OEI patients.

### 2.3 Single‐cell Atlas of Follicular Fluid from OEI Patients Decoded by scRNA‐seq

**Figure 2:**
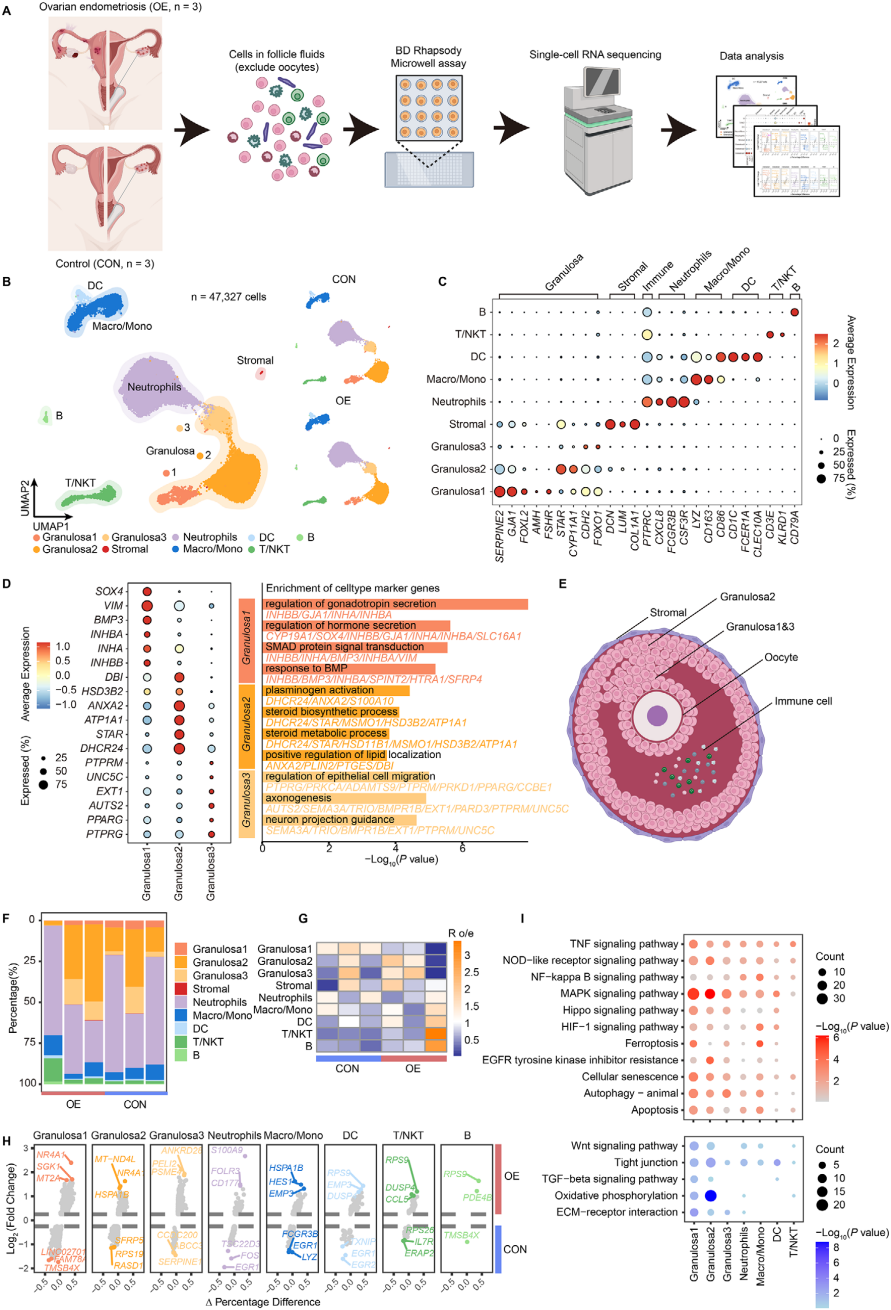
Single‐cell landscape in follicular fluid of patients with OEI. A) Schematic diagram of experimental design. Follicular fluid collected by follicle puncture during IVF/ICSI is subjected to single‐cell RNA sequencing (scRNA‐seq) using the Microwell assay of BD Rhapsody. B) Uniform manifold approximation and projection (UMAP) of scRNA‐seq data from follicular fluid of patients with OEI (n = 3, OE) and patients with male factor infertility (n = 3, CON) (left), and UMAP plot of scRNA‐seq data of the two groups, respectively (right). Colors indicate the major cell types identified in scRNA‐seq. C) Dot plot annotates the average expression level and frequency of representative markers of nine major cell types in scRNA‐seq. D) Expression levels of top markers of three different granulosa cell subpopulations (left) and representative Gene Ontology (GO) terms of different subpopulations (right). E) Schematic diagram shows the simulated niche of major cell types in preovulatory follicular fluid. F) Proportional composition of major cell types in each sample. (G) Heatmap shows the ratio estimate of observed to expected cell numbers with the chi‐square test (R o/e) for each cell types in different samples. H) Differentially expressed genes (DEGs, |Log_2_(Fold change) |>0.25 and adjusted *p* value <0.05) in each cell type between the OE and CON groups. Above the dashed line represents up‐regulation in the OE group, and below the dashed line represents down‐regulation in the OE group. The top three upregulated and downregulated genes in each cell type are labeled. I) Representative KEGG pathways of upregulated DEGs (upper) and downregulated DEGs (lower) are compared between the OE and CON groups in seven cell types. *P* values are calculated by Fisher's exact test.

We performed scRNA‐seq on follicular fluid cells from OEI patients and those undergoing IVF/ICSI due to male factor infertility (**Figures** **2**A, S2A and Table S9, Supporting Information). After quality control and batch effects correction, 47,327 cell transcriptomes were retained for further analysis (Figure S2B–D, Supporting Information). Seven major cell populations were identified based on marker genes^[^ ^15^ ^]^: granulosa cells (*SERPINE2*, *STAR*, *CDH2*), neutrophils (*CSF3R*, *PCGR3B*), monocytes/macrophages (*LYZ*, *CD163*, *CD86*), dendritic cells (*CD1C*, *FCER1A*, *CLEC10A*), T/NKT cells (*CD3E*), B cells (*CD79A*), and a small number of stromal cells (*DCN*, *COL1A1*) (Figure 2B,C, Figure S2E and Table S10, Supporting Information). We validated the accuracy of cell clustering through Gene Ontology (GO) enrichment on the top 50 expressed genes in each cell type to define their biological functions (Figure S2F, Supporting Information) and used SCENIC^[^ ^30^ ^]^ to infer transcription factor (TF) activity (Figure S2G and Table S11, Supporting Information). Unsupervised clustering of granulosa cells identified three subpopulations in uniform manifold approximation and projection (UMAP) space (Figure 2B). Granulosa1, enriched with genes regulating follicle development (e.g., *SOX4*, *INHBA*), is involved in hormone secretion regulation and BMP/SMAD signaling^[^ ^31^ ^]^ (Figure 2D). Granulosa2 is associated with steroid lipid metabolism, while Granulosa3 is linked to epithelial cell migration and projection guidance (Figure 2D). Notably, Granulosa3 cells exhibited a lower number of detected genes and read counts compared to Granulosa1 and 2 (Figure S2H, Supporting Information). Based on these findings, we constructed a schematic representation of follicular cell distribution (Figure 2E). We hypothesize that Granulosa1 corresponds to cumulus granulosa cells, Granulosa2 to mural granulosa cells responsible for hormone synthesis and signaling regulation,^[^ ^32^, ^33^ ^]^ and Granulosa3 to cells involved in cell migration.

We observed significant heterogeneity in cell type proportions across samples (Figure 2F), with Granulosa1 notably enriched in the CON group compared to the OE group (Figure 2G). Comparative analysis of gene expression patterns and TF activities across different cell types between OE and CON groups revealed that *NR4A1* was significantly upregulated in Granulosa 1 and 2 in the OE group, while *EGR1* expression was markedly reduced in the myeloid cell (Figure 2H, Figure S2G and Tables S12 and S13, Supporting Information). NR4A1 overexpression in granulosa cells has been shown to inhibit proliferation and promote apoptosis,^[^ ^34^ ^]^ while EGR1 suppresses inflammatory enhancers in macrophages, reducing their activity and immune response.^[^ ^35^ ^]^ Furthermore, enrichment analyses showed upregulated pathways in the OE group associated with ferroptosis, cellular senescence, apoptosis, and inflammatory pathways, including TNF, NOD‐like receptor, and NF‐κB signaling (Figure 2I and Table S14, Supporting Information). Downregulated pathways included oxidative phosphorylation, ECM‐receptor interaction, tight junctions, Wnt, and TGF‐β signaling. In summary, these data provide a single‐cell atlas of pre‐ovulatory follicular fluid, defining granulosa cell subpopulations’ functions and uncovering unique transcriptional characteristics in the follicular fluid of OEI patients.

### 2.4 Single‐Cell Dynamics in the Iron‐Overloaded Follicular Fluid of OEI Patients

**Figure 3:**
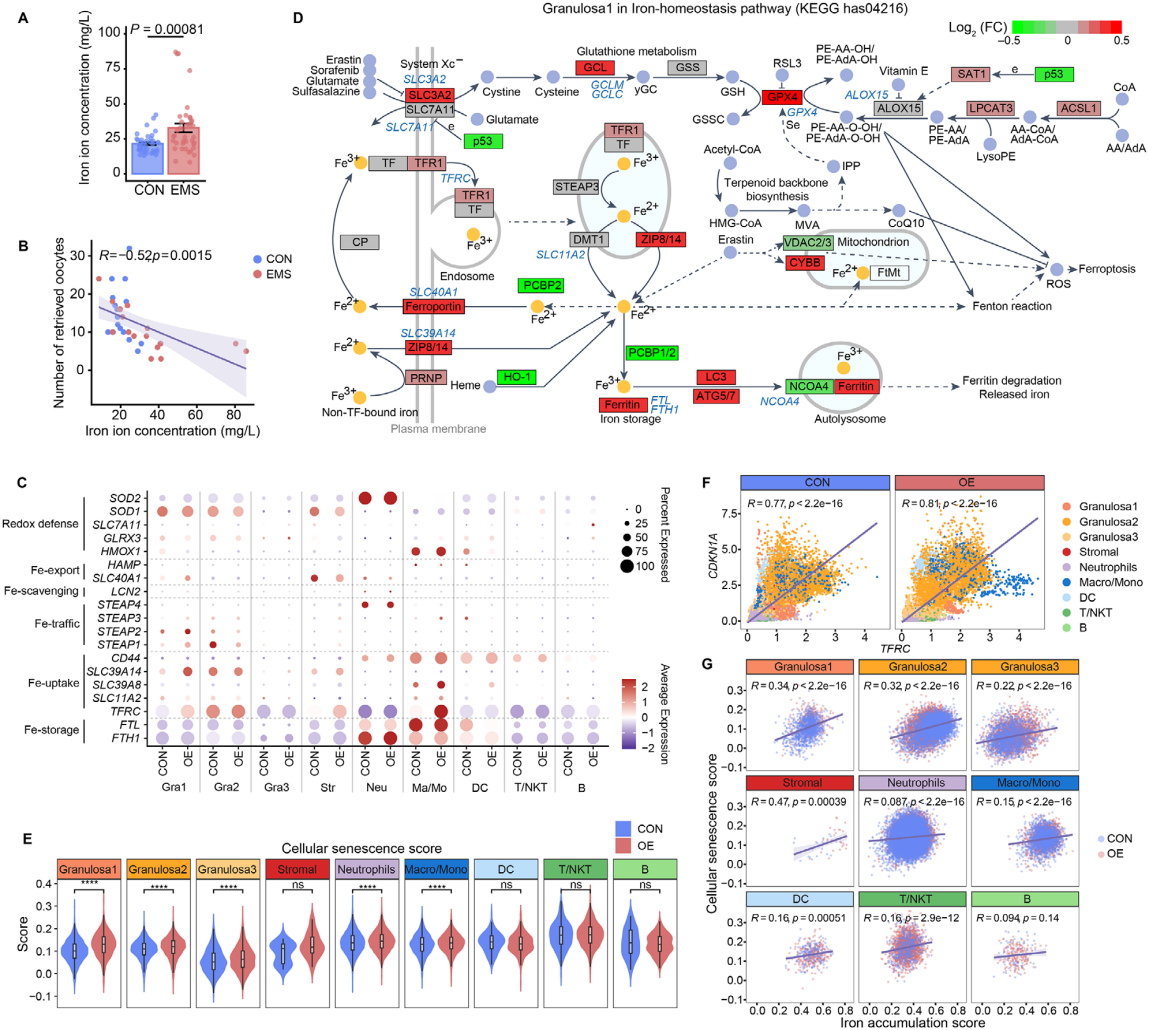
Single‐cell dynamics of iron accumulation in follicular fluid. A) Iron ion levels in follicular fluid of patients with endometriosis (EMS) and non‐endometriosis (CON). Two‐sided Wilcoxon rank‐sum test. B) Pearson correlation analysis between iron ion levels in follicular fluid and the number of retrieved oocytes in corresponding patients. Points are colored by different groups. C) Dot plot shows the average expression and percentage of all cell types expressing genes regulating iron homeostasis. D) Schematic diagram of iron homeostasis pathway (KEGG has 04216), in which genes are colored according to Log_2_(Fold Change) of Granulosa1 in the OE group compared to the CON group. E) Gene set score analysis of cellular senescence pathways of different cell types between the two groups. ****, *p* value < 0.0001; ns, no significance. F) Pearson correlation analysis between *TFRC* and *CDKN1A* gene expression levels of all cell types in follicular fluid scRNA‐seq data. Gene expression matrix is imputed by MAGIC. Each color represents a different type of cell. G) Pearson correlation between iron accumulation score and cell senescence score of different cell types. Each color represents a different group.

Iron overload in follicular fluid has been identified as a critical factor contributing to poor oocyte quality in OEI patients.^[^ ^11^, ^12^, ^36^ ^]^ We measured iron levels in follicular fluid from both endometriosis‐associated and other infertility patients, revealing significantly elevated iron levels in the former group (**Figure** **3**A). These elevated levels negatively correlated with the number of oocytes retrieved (Figure 3B), suggesting that excess iron may impair follicular development and oocyte maturation.

Subsequently, using a curated iron accumulation gene set,^[^ ^37^ ^]^ we confirmed that most cell types in the OE group exhibited a higher iron accumulation signature compared to the CON group, as shown by scRNA‐seq data (Figure S3A, Supporting Information). We further explored the impact of chronic iron overload on various cell types in OEI follicular fluid by assessing the expression of key iron metabolism genes. Granulosa cells and macrophages in the OE group showed increased expression of iron‐uptake genes, including *TFRC*, *SLC39A14*, and *SLC39A8* (Figure 3C and Figure S3B, Supporting Information). Additionally, *SLC40A1*, encoding only iron exporter, was upregulated in the Granulosa1 cluster, indicating iron metabolism abnormality in this cell population of OE group (Figure 3C). Further investigation of the Granulosa1 cluster, as a supporter to oocyte, revealed overexpression of genes involved in the glutathione peroxidase pathway, including *SLC3A2*, *GCLC*, *GCLM*, and *GPX4*, essential for defending against ROS‐mediated lipid peroxidation (Figure 3D). Interestingly, both ferroptosis‐promoting and ‐inhibiting genes were highly expressed in the OE group (Figures S3C,D and Table S6, Supporting Information), with a positive correlation between their expression patterns (Figure S3E, Supporting Information). This suggests that while ferroptosis pathways are activated under iron overload, the cellular antioxidant system may prevent it from occurring.

Recent studies have linked chronic intracellular iron accumulation to cellular senescence.^[^ ^37^, ^38^ ^]^ Consistent with this, our scRNA‐seq data showed increased cellular senescence and senescence‐associated secretory phenotype (SASP) in the follicular fluid of OEI patients (Figure 3E and Figure S3F, Supporting Information). Notably, *TFRC* showed an abnormal positive correlation with the senescence marker *CDKN1A* (Figure 3F). In granulosa cell, iron accumulation was closely associated with cellular senescence (Figure 3G). Our findings, corroborated by external RNA‐seq data, indicate a dynamic imbalance of iron homeostasis in OEI follicular fluid, with iron accumulation potentially driving cellular senescence (Figure S3G, Supporting Information).

In addition, we compared the effects of follicular fluid from control subjects and OEI patients on the granulosa cell line KGN, and observed increased senescence‐associated β‐galactosidase (SA‐β‐gal) activity in cells treated with OEI follicular fluid (Figure S4A, Supporting Information). Direct treatment of KGN cells with ferric ammonium citrate similarly elevated SA‐β‐gal activity, whereas co‐treatment with deferoxamine (DFO) attenuated this effect (Figure S4B, Supporting Information). Additionally, we measured the levels of key senescence‐associated secretory phenotype (SASP) factors, including MCP‐1, IL‐1β, IL‐6, IL‐8, and GM‐CSF in follicular fluid. These cytokines were significantly elevated in endometriosis patients compared to controls, and their levels positively correlated with iron concentration in the FF, further supporting the link between iron overload and a senescence‐associated inflammatory microenvironment in OEI (Figures S4C–G, Supporting Information). Collectively, these findings indicate that excess iron in the follicular fluid of OEI patients directly contributes to senescence.

### 2.5 Metabolic Reprogramming of Cumulus Granulosa Cells in OEI Patients

**Figure 4:**
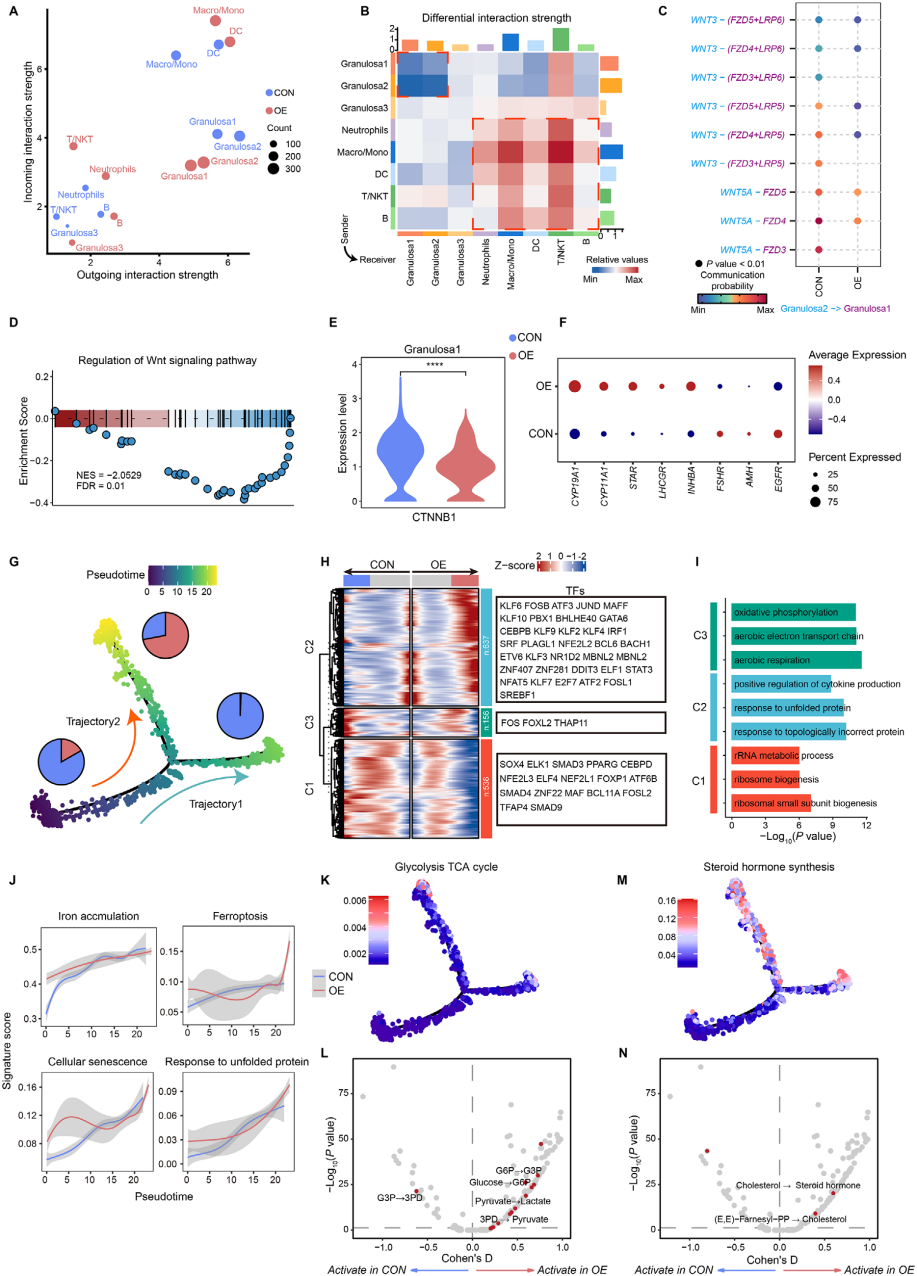
Metabolic reprogramming of Granulosa1 in follicular fluid of patients with OEI. A) Potential outgoing and incoming interaction intensities of each cell type analyzed by CellChat. Different groups are colored differently. B) Heatmap depicts the differentially expressed intensity of the inferred cell communication network. Red (or blue) indicates increased (or decreased) signaling in the OE group compared to the CON group. The colored bars at the top and right indicate the sum of columns (incoming signaling) and rows (outgoing signaling), respectively. C) Bubble heatmap shows cell‐cell communication of selected ligand‐receptor pairs. Dot size indicates *p* value and is colored by communication probability. (D) GSEA of WNT signaling pathway regulation on the OE and CON groups in Granulosa1 cluster. E) Comparison of *CTNNB1* gene expression levels between the two groups in Granulosa1 cluster. Two‐sided Wilcoxon rank sum test. ****p < 0.0001. F) Dot plot shows the average expression and percentage of genes expressing hormone regulation and follicle maturation within Granulosa1 cluster between the two groups. G) Monocle trajectory inference of Granulosa1 cluster cells, colored by their corresponding pseudotime. Pie charts represent the proportion of cells of the two groups in different branches. H) Heatmap shows gene expression along the differentiation trajectory of Granulosa1 of the two groups. Transcription factors (TF) expressed in each branch are listed on the right. I) Bar graph shows the enriched representative GO terms of clustered genes in (H). *p* values are calculated by one‐sided hypergeometric test. J) Expression dynamics of representative gene sets along the pseudotime axis in different groups. K,M) Distribution of metabolic fluxes of glycolysis TCA cycle (K) and steroid hormone synthesis (M) on Monocle pseudotime trajectory. L,N) Volcano plots of representative differential metabolic fluxes in glycolysis (L) and steroid hormone synthesis (N). The abscissa is the Cohen's D effect size. *P* values are determined by Likelihood Ratio test.

From early folliculogenesis to ovulation, oocyte and follicular growth rely on the coordinated interaction between various cell types.^[^ ^18^, ^22^ ^]^ This led us to investigate cell‐cell communication within the follicular ecosystem. In both the OE and CON groups, ligand‐receptor interactions were predominantly driven by myeloid cells, particularly monocytes/macrophages (**Figures** **4**A and S5A, Supporting Information). Moreover, the OE group exhibited stronger immune cell communication but reduced granulosa cell communication compared to the CON group (Figure 4A,B). Notably, granulosa cell subpopulations showed weakened ligand‐receptor interactions, while immune cell interactions intensified (Figure 4B), with activation of inflammatory pathways like MHC, CXCL, and IL10, and a reducing follicle development‐related pathway such as WNT and EGF (Figure S5B, Supporting Information). The inflammatory milieu in OEI follicles may impair the supportive role of granulosa cells in oocyte development.

Interestingly, WNT pathway ligands *WNT3* and *WNT5A* were predominantly expressed in mural‐like Granulosa2, with their receptors mainly in cumulus‐like Granulosa1 (Figure S5C, Supporting Information). Ligand‐receptor analysis revealed a significant reduction in WNT signaling in the OE group (Figure 4C), with a notable decrease in *WNT3* and *WNT5A* expression in Granulosa2 cells (Figure S5D, Supporting Information). Downstream WNT signaling was also significantly inhibited in Granulosa1 cells in the OE group (Figure 4D), including reduced *CTNNB1* expression (Figure 4E), which encodes β‐catenin, a core component of the WNT pathway. Previous studies have shown that WNT pathway activation regulates granulosa cell proliferation and steroidogenesis.^[^ ^39^ ^]^ Dysregulation of the WNT pathway in OEI follicles may impair oocyte maturation and hormone metabolism, as evidenced by the upregulation of steroidogenesis‐related genes (*CYP19A1, CYP11A1, STAR*) and downregulation of genes promoting oocyte maturation (*FSHR, AMH, EGFR*) in Granulosa1 cells (Figure 4F).

Next, we applied Monocle^[^ ^40^ ^]^ to infer the differentiation trajectory of Granulosa1 cells, exploring the dynamics of cumulus cell fate under iron‐overloaded conditions. The trajectory initially overlapped but diverged into two main branches: Branch 1 contained predominantly CON cells, while Branch 2 consisted mainly of the OE (Figure 4G and Figure S5E, Supporting Information). Differential gene expression along these branches highlighted key TFs (Figure 4H). GO enrichment analysis revealed that in the OE group, senescence‐related processes, including cytokine production and the response to unfolded/misfolded proteins, were activated, while pathways related to oxidative phosphorylation and mitochondrial aerobic respiration were activated in the CON group (Figure 4I). Gene signature related to iron accumulation, ferroptosis, cellular senescence, and the unfolded protein response increased along the pseudo‐time axis, with consistently higher scores in the OE group (Figure 4J), indicating a senescence‐associated phenotype in OEI cumulus granulosa cells linked to mitochondrial respiratory dysfunction induced by iron accumulation. To further explore this connection, we assessed mitochondrial function under iron‐overloaded conditions. We observed a significant decrease in mitochondrial membrane potential, along with markedly elevated mitochondrial ROS levels (Figure S6, Supporting Information). These alterations are hallmarks of mitochondrial dysfunction and are known to trigger cellular senescence. Importantly, treatment with DFO effectively rescued both the loss of mitochondrial membrane potential and the ROS overproduction (Figure S6, Supporting Information), thereby confirming the causative role of iron accumulation in mediating these changes.

To further characterize the metabolic shifts in OEI cumulus granulosa cells, we used scFEA^[^ ^41^ ^]^ and scMetabolism^[^ ^42^ ^]^ to assess changes in metabolic flux between the OE and CON groups (Table S15, Supporting Information). While overall glycolysis TCA cycle flux increased in the OE group, there was an enhancement in glycolysis/gluconeogenesis pathways and a reduction in TCA cycle activity (Figure 4K and Figure S5F,G, Supporting Information), particularly with increased flux in the lactate production (Figure 4L). Additionally, hypoxia‐inducible factor *HIF1A* expression was elevated in most cell types (Figure S5H, Supporting Information). These findings suggest that the iron‐overloaded environment in OEI patients induces hypoxia, leading to the activation of anaerobic respiration and disruption of energy metabolism. Consistent with these findings (Figure 4F), steroid hormone biosynthesis pathways were upregulated along the pseudo‐time axis in the OE group (Figure 4M,N and Figure S5I, Supporting Information), and cohort data showed significantly elevated serum estrogen levels in OEI patients (Figure S5J, Supporting Information). Overall, these results highlight the hypoxia‐induced metabolic reprogramming in cumulus granulosa cells under iron overload in OEI patients, may leading to excessive hormone production and impaired oocyte maturation.

### 2.6 Monocyte/Macrophage Landscape in Iron‐Overloaded Follicular Environment

**Figure 5:**
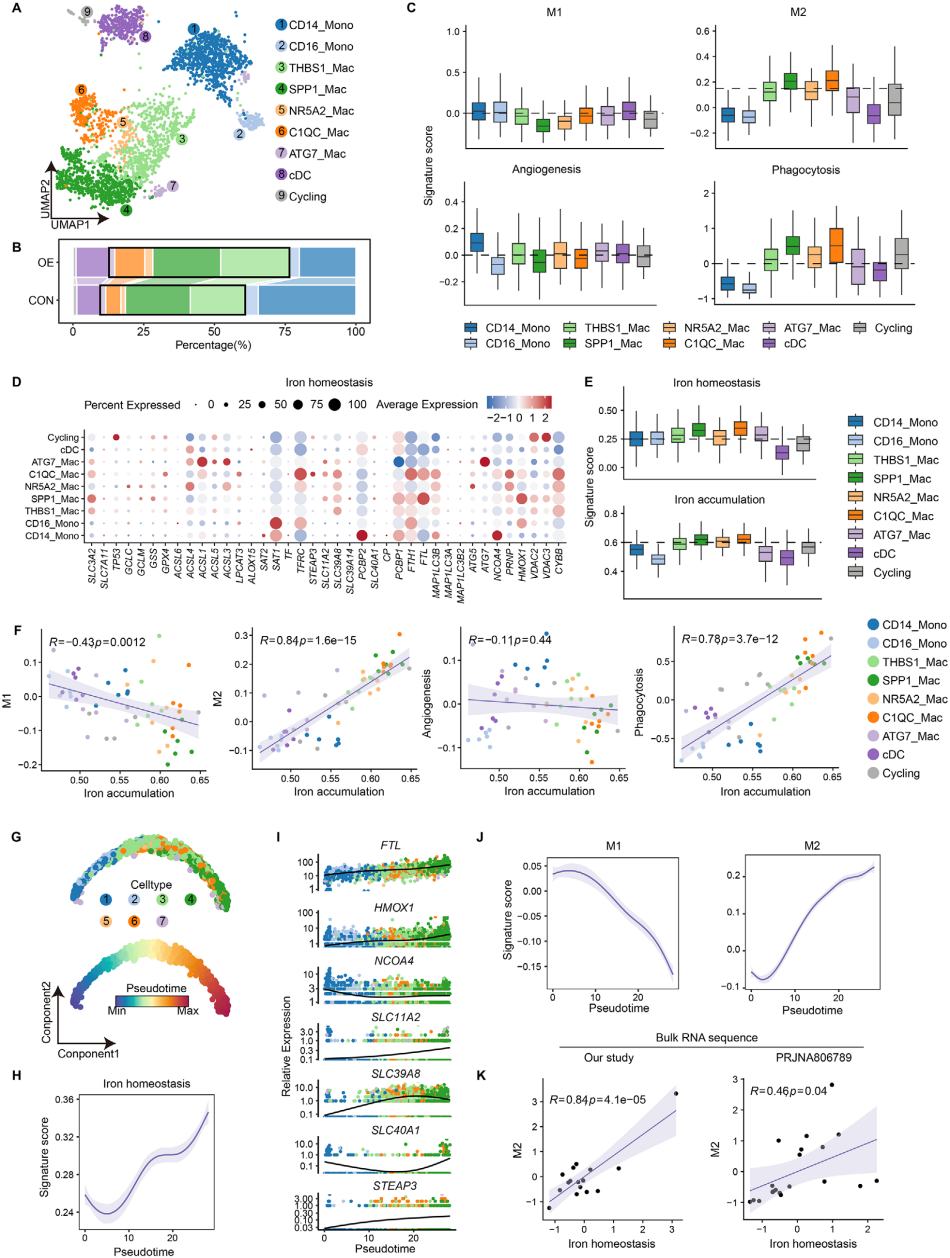
Relationship between iron metabolism and M1/M2 phenotype in myeloid cell subsets of follicular fluid. A) UMAP view of myeloid cell clusters in follicular fluid. B) Proportional composition of myeloid cell subsets in OE group and CON group. The macrophage subcluster is marked by a black box. C) Expression levels of M1‐like, M2‐like, angiogenic, and phagocytic signature genes across myeloid cell clusters. D) Expression levels and frequencies of genes in the iron homeostasis across the myeloid cell cluster. E) Expression levels of iron homeostasis (top) and iron accumulation (bottom) gene signatures in myeloid cell clusters. F) Pearson correlation between iron accumulation signature and M1‐like, M2‐like, angiogenic, and phagocytic signature genes in myeloid cell subclusters in samples. Points represent each cell subcluster in each sample and are colored by different myeloid cell subclusters. G) Monocle trajectory inference of myeloid cells (top), colored by their corresponding pseudotime (bottom). H,J) Expression dynamics of iron homeostasis (H), M1‐like (J, left) and M2‐like (J, right) signature along the pseudotime axis. I) Expression dynamics of iron homeostasis representative genes along the pseudotime. Each point represents the gene expression level in each cell and is colored by different subpopulations. K) Pearson correlation between iron homeostasis score and M2 gene signature in two follicular fluid bulk RNA sequencing data.

We observed that monocyte/macrophages are highly responsive to iron overload, with the OE group exhibited increased expression of iron‐uptake genes and the ferroptosis‐sensitive gene *HMOX1* (Figure 3C). This was accompanied by elevated signatures for iron accumulation, ferroptosis drivers, and inhibitors (Figure S3C,D, Supporting Information). Then, we analyzed the transcriptional dynamics of monocyte/macrophages under iron‐overloaded conditions, identifying seven states, including two monocyte clusters and five macrophage clusters (**Figures** **5**A and S7A,B, Supporting Information). Macrophages were more abundant in the OE group, particularly the C1QC_Mac subtype (Figure 5B and Figure S7C, Supporting Information). Macrophage functional analysis^[^ ^43^ ^]^ that SPP1_Mac and C1QC_Mac macrophage clusters expressed high levels of M2‐like and phagocytosis‐related genes, while M1‐like genes were expressed at lower levels (Figure 5C and Figure S7D, Supporting Information). Additionally, we noted that *LYVE1*, a marker of tissue‐resident macrophages,^[^ ^44^ ^]^ was expressed in the C1QC_Mac, NR5A2_Mac, SPP1_Mac, and THBS1_Mac clusters (Figure S7D, Supporting Information). The C1QC_Mac cluster, characterized by high *FN1* expression, and the SPP1_Mac cluster, marked by high *SPP1* expression, have been identified as pro‐fibrotic macrophage subtypes.^[^ ^45^ ^]^ These findings suggest an M2‐like immunosuppressive environment dominated by C1QC_Mac and SPP1_Mac macrophages in OEI patients’ follicular fluid.

Next, we assessed iron accumulation and homeostasis across monocyte/macrophage clusters, finding that major macrophage subclusters exhibited higher expression of iron homeostasis and accumulation genes than monocytes (Figure 5D,E). Both the C1QC_Mac and SPP1_Mac clusters, which highly express M2‐like genes, also showed high expression of iron storage proteins *FTH1* and *FTL* (Figure 5C,D). Clusters with high iron metabolism scores were strongly correlated with M2‐like and phagocytosis gene expression but inversely correlated with M1‐like gene expression (Figure 5F and Figure S7E, Supporting Information).

Interestingly, the C1QC_Mac cluster highly expressed *TFRC*, while the SPP1_Mac cluster exhibited elevated *HMOX1* expression (Figure 5D), indicating that distinct mechanisms of iron absorption. Additionally, iron homeostasis and senescence marker *CDKN1A* expression were higher across most monocyte/macrophage clusters in the OE group compared to the CON group (Figure S7F,G, Supporting Information), suggesting that robust iron metabolism and senescence characteristics in macrophages.

Monocle trajectory analysis revealed a linear differentiation pathway for monocyte/macrophages, starting from CD14_Mono and C16_Mono clusters and progressing through THBS1_Mac and NR5A2_Mac to terminal SPP1_Mac and C1QC_Mac states (Figure 5G and Figure S7H, Supporting Information). GO enrichment analysis indicated that initial monocytes responded to steroid hormone stimuli, possibly activating the MAPK cascade to promote macrophage differentiation. Differentiated macrophages responded to immune stimuli, engaging in oxidative phosphorylation and ATP synthesis, while terminal macrophages participated in iron metabolism‐related lipoprotein particle responses (Figure S7I, Supporting Information). Iron homeostasis and accumulation gene expression increased along the pseudo‐time trajectory (Figure 5H and Figure S7J, Supporting Information), with upregulation of genes related to iron storage (*FTL*), absorption (*SCL11A2, SLC39A8*), export (*SLC40A1*), transport (*STEAP3, SLC40A1*), and antioxidation (*HMOX1*), while ferritinophagy‐related gene *NCOA4* gradually decreased (Figure 5I). M2‐like gene expression increased along the pseudo‐time axis, whereas M1‐like gene expression showed the opposite trend (Figure 5J). RNA‐seq of follicular fluid also showed a strong correlation between iron metabolism and M2‐like gene expression (Figure 5K and Figure S7K, Supporting Information). These findings highlight the role of macrophages with high iron metabolism in establishing an M2‐like immunosuppressive environment in follicular fluid. Finally, we identified a potential correlation between M1/M2 polarization and estrogen levels (Figure S7L, Supporting Information).

### 2.7 High‐Resolution Spatial Transcriptomics of Iron‐Overloaded Mouse Ovaries

**Figure 6:**
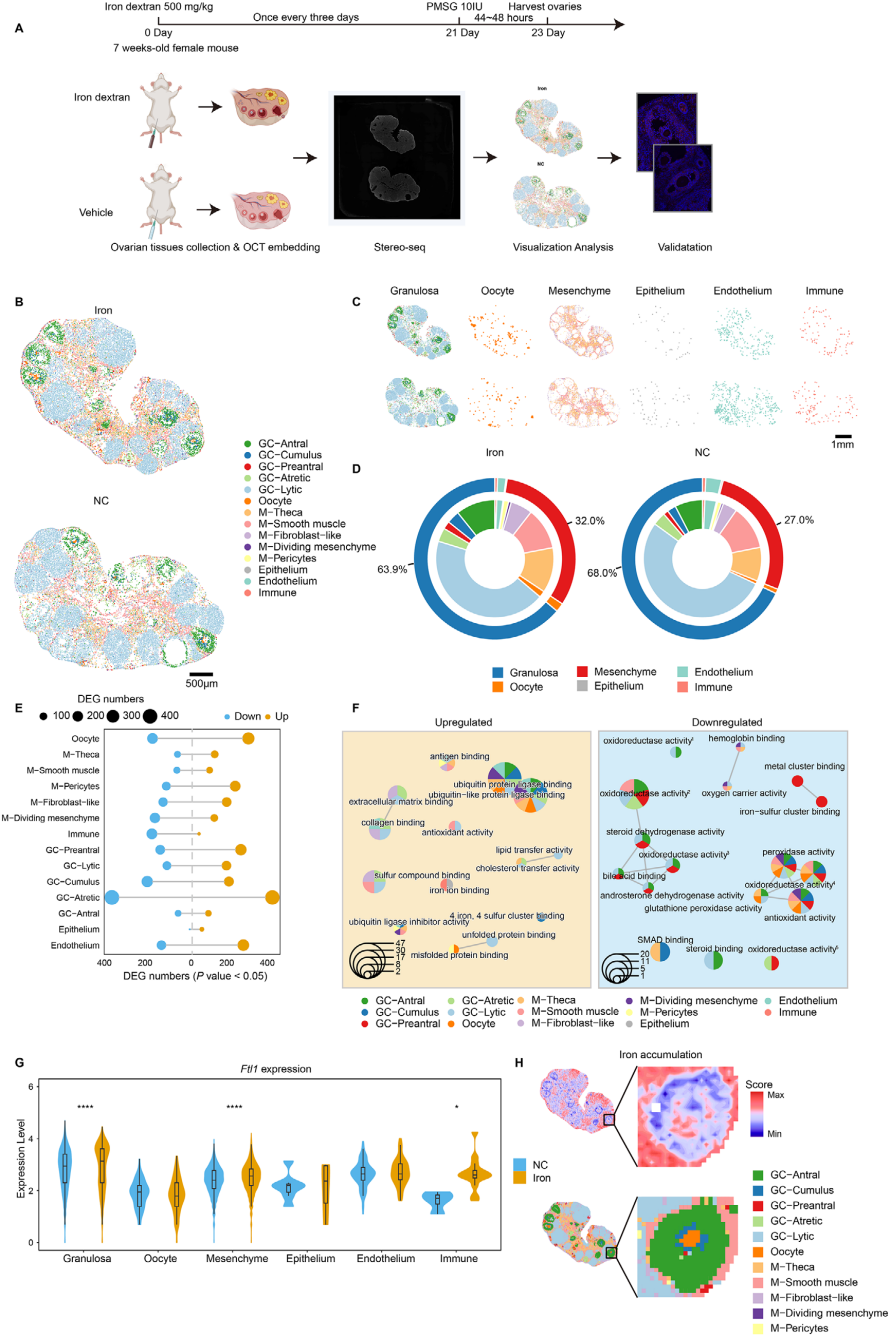
Construction of high‐resolution spatial transcriptome atlas of iron‐overloaded mouse ovaries. A) Schematic diagram of the construction of a mouse iron‐overloaded ovary model and the Stereo‐seq spatial transcriptome analysis. B) Spatial visualization of cell type distribution by RCTD deconvolution in Stereo‐seq data. Scale bars, 500 µm. C) Visualization of the spatial distribution of six major cell types. Colored by subdivided cell types. Scale bars, 1mm. D) Sector diagram shows the proportion of cell types in iron‐overloaded ovaries (Iron) and negative control (NC) ovaries. The outer circle represents the proportion of six major cell types, and the inner circle represents the proportion of all cell types. E) Number of DEGs of all cell types in Iron and NC ovaries in Stereo‐seq data. F) Network visualization of representative GO terms and pathways of DEGs of different cell types. Nodes represent GO terms. Pie charts show the proportion of the number of genes reaching a certain term in spot types. oxidoreductase activity^[^ ^1^ ^]^: acting on paired donors, with incorporation or reduction of molecular oxygen, reduced iron‐sulfur protein as one donor, and incorporation of one atom of oxygen. oxidoreductase activity,^[^ ^2^ ^]^ acting on CH−OH group of donors. oxidoreductase activity,^[^ ^3^ ^]^ acting on NAD(P)H. oxidoreductase activity,^[^ ^4^ ^]^ acting on peroxide as acceptor. oxidoreductase activity,^[^ ^5^ ^]^ acting on a heme group of donors. G) Expression levels of *Ftl1* gene in six major cell types compared in the two groups. Two‐sided Wilcoxon rank sum test. ****p < 0.0001; *p < 0.05. H) Visualization of iron accumulation gene signature zoning layer in the spatial transcriptome of Iron ovaries. Enlarged antral follicle area of iron accumulation signature zoning layer (upper right), and spatial distribution of cell subpopulations (down right).

Building on our above findings, we aimed to explore the transcriptional dynamics of various cell types in the whole ovary under iron‐overloaded conditions. We modeled mouse iron‐overloaded ovaries using a previously reported method^[^ ^10^, ^11^ ^]^ and mimicked the superovulation protocol of IVF/ICSI patients by administering PMSG intraperitoneally 48 h before tissue collection (**Figure** **6**A). We then employed Stereo‐seq, a high‐resolution (220 nm/spot) spatial transcriptomics technique, on freshly frozen, OCT‐embedded ovarian tissues from both iron‐overloaded (Iron) and negative control (NC) mouse ovaries (Methods).

After initial quality control (Figure S8A,B, Supporting Information), we generated 25,636 high‐resolution spatial transcriptomes. Deconvolution and cell‐type assignment, using scRNA‐seq references,^[^ ^22^ ^]^ revealed six broad cell types: granulosa cells, mesenchymal cells, oocytes, endothelial cells, epithelial cells, and immune cells (Figure 6B–D and Table S16, Supporting Information). This dataset provides a near‐single‐cell resolution spatial transcriptomic map of mouse ovaries. Granulosa cell classification identified mural granulosa cells along the follicle wall and cumulus cells surrounding the oocyte, with further clustering revealing four mural granulosa subtypes associated with different follicle states: preantral, antral, atretic, and luteinizing (Figure 6B,C and Figure S8C, Supporting Information). Additionally, we deconvoluted markers of mesenchymal cell types and identified smooth muscle cells, fibroblast‐like cells, theca cells, proliferating mesenchymal cells, and pericytes (Figure 6B,C and Figure S8D, Supporting Information). Notably, the Iron group exhibited a decrease in granulosa cells and an increase in mesenchymal cells (Figure 6D), suggesting that iron overload promotes ovarian fibrosis, which was further confirmed by Picrosirius Red staining (Figure S8F, Supporting Information).

To spatially resolve the transcriptomic features of iron‐overloaded ovaries, we identified differentially expressed genes (DEGs) between the Iron and NC groups across different cell types (Figure 6E, Figure S8E and Table S17, Supporting Information). Overall, in atretic follicle granulosa cells, oocytes, and cumulus granulosa cells, the largest number of DEGs were found (Figure 6E), indicating cell state‐specific transcriptional effects under iron overload. Functional enrichment analysis revealed upregulation of ubiquitin and ubiquitin‐like protein ligase pathways in most cell types, with granulosa cells showing heightened iron metabolism and mesenchymal subclusters upregulating collagen and extracellular matrix binding pathways (Figure 6F). And certain subgroups displayed increased antioxidant activity, lipid transfer, and unfolded protein pathways. Downregulated pathways included antioxidant and oxidoreductase activities, highlighting oxidative damage in the ovarian microenvironment and the unique antioxidant transcriptional characteristics of different cell subgroups under iron overload (Figure 6F).

We further explored the sensitivity of ovarian cell types to iron to evaluate the potential for targeted cell‐type‐specific therapies in OEI patients. *Ftl1* gene was significantly upregulated in granulosa cells, mesenchymal cells, and immune cells in the Iron ovaries, with pronounced expression in luteinized granulosa cells, smooth muscle cells, and fibroblast‐like cells (Figure 6G and Figure S8G, Supporting Information). Spatial distribution analysis using the iron accumulation score from the Stereo‐seq data showed that mesenchymal cell subgroups and some luteinized granulosa cells had high iron accumulation, confirmed by Prussian blue staining (Figure 6H, Figure S8H and Table S18, Supporting Information). Interestingly, upon zooming into the antral follicle region, cumulus granulosa cells exhibited high iron accumulation, suggesting a compensatory role for excess iron in oocytes (Figure 6H).

Finally, spatial transcriptomic data validated the ecological distribution of granulosa cell subgroups identified in single‐cell data from follicular fluid (Figure S8I, Supporting Information). Cumulus‐like Granulosa1 signature was mainly expressed within the antral follicle and around the oocyte, mural‐like Granulosa2 in the post‐ovulatory corpus luteum, and corona radiata‐like Granulosa3 within the antral follicle, encircling a larger Granulosa1 cluster. These findings emphasize the accuracy of our granulosa cell subgroup definitions in follicular fluid.

### 2.8 Senescence Characteristics in Iron‐Overloaded Mouse Ovaries

**Figure 7:**
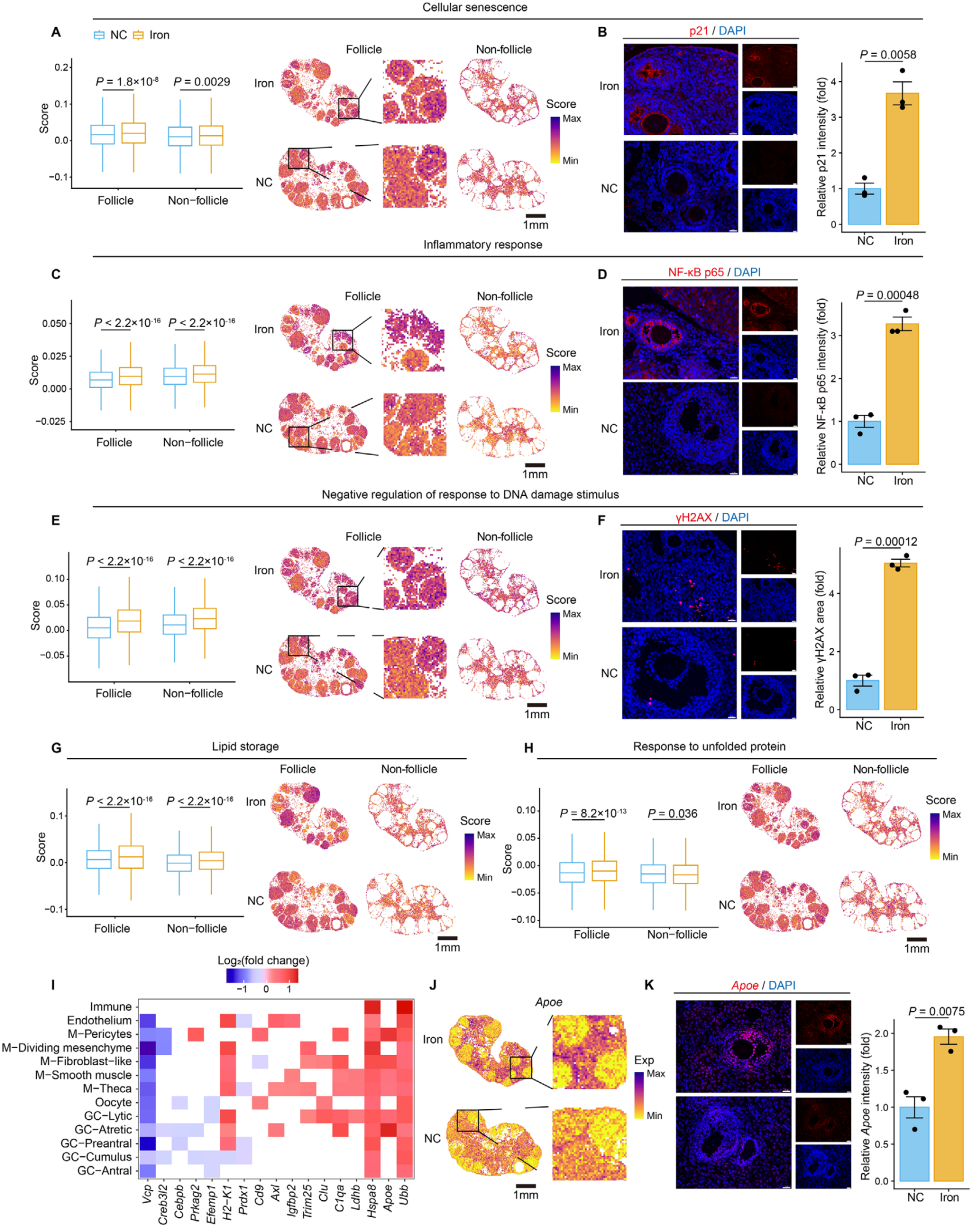
Transcriptional changes of typical senescence markers in iron‐overloaded ovaries. A, C, E, G, H) Box plots (left) and spatial visualizations (right) show the global distribution density of gene signatures of cellular senescence (A), inflammatory response (C), negative regulation of response to DNA damage stimulus (E), lipid storage (G), and response to unfolded protein (H) in iron‐overloaded ovaries (Iron) and negative control ovaries (NC). Scale bars, 1mm. B, D, F) Immunofluorescence staining of p21 (B), NK‐κB p65 (D), and γH2AX (F) in Iron and NC ovaries (left). Scale bars, 20µm. Relative intensities are quantified as fold changes and are represented on the right as mean ± SEM. n = 3 for each group. Two‐tailed Student's *t*‐test. I) Heatmap shows genes that are upregulated and downregulated DEGs identified in at least three cell types overlapping with the aging atlas database. J) Spatial visualization of *Apoe* gene expression level. Scale bars, 1 mm. K) Immunofluorescence staining of *Apoe* in Iron and NC ovaries (left). Scale bars, 20µm. Relative intensities are quantified as fold changes and are represented on the right as mean ± SEM. n = 3 for each group.

Following our findings of senescence characteristics in the follicular fluid of OEI patients with iron overload (Figure 3E–H and Figure S3A,F, Supporting Information), we investigated senescence features in iron‐overloaded mouse ovaries. First, we assessed the spatial distribution of cellular senescence gene signatures in NC and Iron ovaries. We observed a significant increase in these signatures within both follicular and non‐follicular regions of iron‐overloaded ovaries, with the cell cycle inhibitors p21 notably upregulated (**Figure** **7**A,B). Chronic inflammation and SASP are tightly linked and collectively drive cellular senescence. In iron‐overloaded ovaries, inflammation‐related gene signatures were markedly upregulated in follicular regions, particularly in the corpus luteum, accompanied by activation of the NF‐κB inflammatory signaling pathway (Figure 7C,D). Given that DNA damage‐induced genomic instability is a hallmark of senescence,^[^ ^46^ ^]^ we further examined the response to DNA damage. The ability to regulate DNA damage response was diminished, and γH2AX‐positive regions, indicative of DNA damage, were significantly increased (Figure 7E,F). Additionally, genes associated with lipid storage were upregulated, particularly in the corpus luteum (Figure 7G). The unfolded protein response in mitochondria, crucial for clearing misfolded proteins, was upregulated in follicular regions but downregulated in non‐follicular regions, suggesting that iron deposition may disrupt the balance between refolding and clearance of aberrant proteins in mesenchymal cells (Figure 7H and Figure S8H, Supporting Information).

Next, we mapped senescence‐related genes expression changes across all cell types under iron overload (Figure S9A, Supporting Information). From Aging Atlas database,^[^ ^47^ ^]^ we identified 82 senescence‐related DEGs, with 17 DEGs found in at least three cell types (Figure 7I). Among these, heat shock protein *Hspa8* and ubiquitin protein *Ubb* were upregulated across all cell types, while ATPase family protein *Vcp* was downregulated in most (Figure 7I and Figure S9B,C, Supporting Information). Additionally, Apolipoprotein E (APOE), a key protein in lipid metabolism reported to be elevated in aging and endometriosis ovaries^[^ ^48^, ^49^ ^]^ and associated with reduced mature oocyte retrieval in older women,^[^ ^50^ ^]^ was identified as a DEG primarily in the corpus luteum, atretic follicles, and mesenchymal cells of iron‐overloaded ovaries (Figure 7I–K). Spatial transcriptomics revealed significant differential expression of *Apoe* in non‐follicular regions (Figure 7J and Figure S9C, Supporting Information).

Finally, we explored transcriptional changes in pre‐ovulatory antral follicle oocytes under iron overload (Figure S9D, Supporting Information). We found downregulation of *Gja4*, a TZP key component, and *Slc39a10*, a zinc transporter, and the upregulation of *Clu*, a marker of apoptotic atretic follicles and ovarian cancer^[^ ^51^, ^52^ ^]^(Figure S9E, Supporting Information). *APOE* expression was notably elevated in oocytes from iron‐overloaded antral follicles and OEI patients (Figure S9E,F, Supporting Information), linking iron overload with senescence and suggesting potential therapeutic implications. Enrichment of apoptotic signaling pathways, inflammatory responses, and downregulation of cell cycle and biological process regulation pathways in iron‐overloaded oocytes indicate aberrant transcriptional programming under iron overload. (Figure S9G, Supporting Information). These findings highlight the activation of senescence‐related transcriptional programs across various cell types in iron‐overloaded ovaries, potentially impacting oocyte development and maturation.

### 2.9 Dynamics of Iron Metabolism Imbalance in Aging Human Ovaries

**Figure 8:**
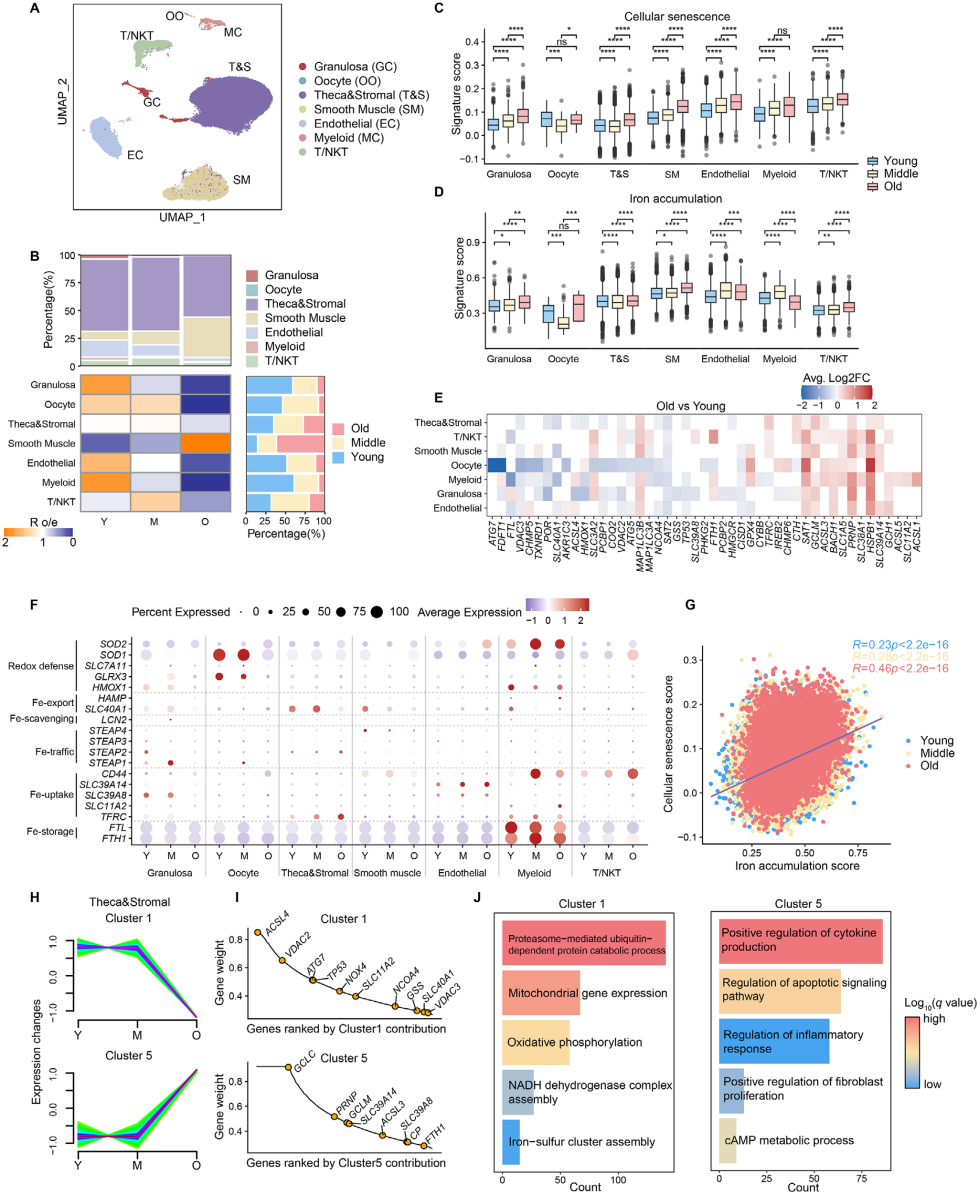
Iron homeostasis profiling in human aging ovarian scRNA‐seq atlas. A) UMAP plots of scRNA‐seq data from ovarian tissues of young (n = 3), middle‐aged (n = 3), and elderly individuals (n = 3), colored by major ovarian cell types. B) Heatmap showing the enrichment of different cell types across age groups, estimated by the ratio of observed to expected cell counts (Ro/e) and analyzed using a chi‐squared test. The top bar chart represents cell type composition, and the right bar chart displays the composition across different age groups. C,D) Gene signature scoring for cellular senescence (C) and iron accumulation (D) across ovarian cell types in the three age groups. Two‐sided Wilcoxon rank‐sum test. ****p < 0.0001; ***p < 0.001; **p < 0.01; *p < 0.05; ns, no significance. E) Heatmap of DEGs related to ferroptosis between the Old and Young groups. Red indicates upregulation in the Old group, while blue indicates downregulation. F) Average expression levels and percentages of cells expressing genes regulating iron homeostasis. G) Pearson correlation between iron accumulation scores and cellular senescence scores across different groups, with each color representing a different group. H) Line plots depicting the age‐related dynamics of standardized gene expression in theca & stromal cells, derived from fuzzy clustering analysis. Green lines indicate the expression trajectories of individual genes. I) Ranking of genes in Cluster 1 and Cluster 5 from (H) based on gene weight (contribution), highlighting genes related to iron homeostasis. J) GO enrichment analysis of Cluster 1 and Cluster 5. *p* values were calculated using the hypergeometric test and corrected for multiple comparisons using the Benjamini‐Hochberg method.

To further investigate the role of iron overload in ovarian aging, we analyzed scRNA‐seq data from ovarian tissues of nine individuals across three age groups: young (18–28 years, Y, n = 3), middle‐aged (37–39 years, M, n = 3), and older adults (47–49 years, O, n = 3), all of whom underwent oophorectomy due to cervical or endometrial cancer.^[^ ^14^ ^]^ Using established ovarian cell markers, we identified seven major cell types (**Figures** **8**A and S10A, Supporting Information). Among these, the proportion of granulosa cells, oocytes, endothelial cells, and myeloid cells exhibited a gradual decline with age, whereas smooth muscle cells showed a significant increase in the older group (Figure 8B). Both cellular senescence and SASP gene signatures became increasingly prominent with age (Figure 8C and Figure S10B, Supporting Information). Notably, iron accumulation gene signatures also showed an age‐dependent increase across most cell types, suggesting age‐related changes in ovarian cell iron homeostasis (Figure 8D).

Differential expression analysis revealed age‐associated alterations in ferroptosis‐related gene sets across various ovarian cell types (Figure 8E, Figure S10C,D and Table S19, Supporting Information). Heat shock protein *HSPB1* and prion protein *PRNP* were upregulated exclusively in older individuals, while *SAT2* expression decreased (Figure 8E and Figure S10C, Supporting Information). Analysis of key iron homeostasis genes revealed marked upregulation of iron storage proteins in myeloid cells, indicating their role in regulating local iron concentration (Figure 8F). Meanwhile, *TFRC* expression increased with age in theca and stromal cells, while antioxidant enzymes *SOD1* and *GLRX3* were significantly downregulated in oocytes of the older group (Figure 8F). Correlation analysis revealed a strong association between iron accumulation gene signatures and both cellular senescence and SASP, particularly in the older group (Figure 8G and Figure S10E, Supporting Information).

Further evaluation of iron metabolism dynamics during ovarian aging revealed three distinct trends: gradual increase, decrease, or biphasic patterns (Figure S10F, Supporting Information), indicating dynamic changes in iron metabolism pathways during aging. Given that fibroblasts contribute to age‐related inflammation and fibrosis through repeated menstrual cycles, potentially causing irreversible ovarian damage,^[^ ^53^ ^]^ we focused on the iron metabolism characteristics of fibroblasts in aging process. Time‐series analysis clustered the transcriptomes of theca and stromal cells into eight unique dynamic patterns (Figure S10G, Supporting Information). Clusters 1 and 5 exhibited significant age‐related changes in gene expression (Figure 8H). In Cluster 1, iron metabolism was characterized by downregulation of ferroptosis‐related genes (*ACSL4*, *VDAC2/3*, *NOX4*), ferritinophagy genes (*ATG7*, *NCOA4*), and iron export genes (*SLC40A1*, *SLC11A2*) in older individuals (Figure 8I). Conversely, Cluster 5 showed upregulation of ferroptosis inhibitors (*ACSL3*), antioxidant defense genes (*GCLC, GCLM*), iron import genes (*SLC39A14*, *SLC39A8*), and ferritin (*FTH1*), indicating increased resistance to ferroptosis and iron accumulation in aging ovarian stromal cells (Figure 8I). GO enrichment analysis revealed reduced mitochondrial function, oxidative‐reduction capacity, and ubiquitin‐dependent protein degradation processes in the older group, alongside activation of inflammation, fibrosis, and cytokine production (Figure 8J). Overall, these findings underscore the dynamic regulation of iron homeostasis genes with aging, coupled with an emerging iron accumulation phenotype in aging ovaries.

## 3 Discussion

Previous studies have reported iron overload in the follicular fluid of OEI patients.^[^ ^11^, ^12^ ^]^ Our study provides a comprehensive comparison of single‐cell atlases of preovulatory follicular fluid in OEI patients and spatial transcriptomic maps of iron‐overloaded mouse ovaries, highlighting differences across cellular ecosystems and molecular characteristics relative to normal controls. Investigating follicular development and ovarian dysfunction under iron overload offers the potential to identify early disease‐associated molecules that could serve as therapeutic targets. The reduced quality of oocytes in OEI patients undergoing IVF/ICSI treatment may result from aberrant interactions among various cell types within the iron‐overloaded environment.

Our findings demonstrate that key cell types in follicular fluid are variably affected by environmental iron, with granulosa cells and macrophages being particularly susceptible. Although cellular antioxidant systems and iron metabolism processes offer some resistance to excessive extracellular iron, iron accumulation persists in damaged or senescent cells.^[^ ^37^ ^]^ Extensive research has shown that excess extracellular iron contributes to fibrotic diseases, such as small vessel injury and iron release, partially explaining the favorable environment for endometriotic lesion growth and invasion in endometriosis patients with retrograde menstruation (which introduces heme‐containing menstrual blood into the peritoneal cavity).^[^ ^54^ ^]^

However, excess intracellular iron can initiate cellular damage and progressively contribute to the development of a senescent phenotype. Our data reveal that in the iron‐overloaded follicular fluid of OEI patients, *TFRC* expression is elevated, correlating with iron accumulation, and that some cells show a strong association between high levels of iron accumulation and the expression of senescence‐related markers. Recent studies indicate that abnormal iron metabolism in aged mouse ovaries and oocytes can be ameliorated by in vivo and in vitro application of iron chelators, which improves ovarian reserve and oocyte iron metabolism.^[^ ^38^ ^]^ This suggests that intracellular iron accumulation may be a key factor in inducing the senescent phenotype and contributing to infertility. Therefore, assessing iron ion levels in the follicular fluid of endometriosis‐related infertility patients may serve as a predictive marker for ART clinical pregnancy rates.

Notably, in the follicular fluid of OEI patients, there is a marked reduction in WNT pathway signaling between cumulus granulosa cells and mural granulosa cells, accompanied by a significant decrease in the expression of the core downstream protein β‐catenin (*CTNNB1*). The conserved WNT/β‐catenin signaling pathway has been shown to regulate follicle development and maturation across various model organisms.^[^ ^55^ ^]^ We further observed downregulation of genes such as *FSHR* and *EGFR*, which are involved in oocyte maturation and ovulation. This suggests that the suppression of WNT/β‐catenin signaling in the iron‐overloaded environment may hinder oocyte maturation and follicle ovulation.^[^ ^56^ ^]^ Another role of cumulus granulosa cells is to supply pyruvate and other branched‐chain amino acids for oxidative phosphorylation in the oocyte.^[^ ^18^ ^]^ However, we found that with increasing levels of intracellular iron accumulation, cumulus cells exhibited enhanced anaerobic glycolysis and produced large amounts of lactate, potentially leading to oocyte meiotic arrest.^[^ ^57^ ^]^ Additionally, changes in metabolic flux in other cell types provide further insight into the metabolic dynamics of iron overload in follicular fluid.

Our results demonstrate that DFO treatment effectively alleviates iron overload‐induced senescence and mitochondrial dysfunction in granulosa cells, supporting its potential as a therapeutic agent. Targeting iron overload may thus represent a promising strategy to improve oocyte quality in OEI patients. Clinically, iron chelators such as DFO have been widely used to treat systemic iron overload and have shown protective effects in reproductive models.^[^ ^11^, ^58^ ^]^ However, their application in OEI poses challenges. Systemic administration may cause adverse effects, including hepatic or renal toxicity, particularly in patients with normal systemic iron levels.^[^ ^58^ ^]^ Moreover, selectively reducing iron levels within the ovarian microenvironment without disrupting systemic iron homeostasis remains difficult. Future studies should explore localized delivery approaches, such as intraovarian administration or nanoparticle‐based systems, to enhance specificity and minimize side effects. Overall, while repurposing iron chelators is a feasible direction, further preclinical evaluation is essential to establish their safety and efficacy in the context of OEI.

Our data indicate that as ovarian aging progresses, the molecular characteristics of iron metabolism undergo distinct dynamic changes. Fibrosis and inflammation, both of which are processes coordinated by fibroblasts, are hallmark features of ovarian aging.^[^ ^53^, ^59^ ^]^ From a transcriptomic perspective, aging ovarian fibroblasts exhibit increased expression of genes involved in iron uptake, decreased expression of genes responsible for iron export, and suppression of ferroptosis‐related genes, consistent with previous reports.^[^ ^60^ ^]^ This suggests that aging fibroblasts may continuously accumulate iron, driving the progression of ovarian inflammation and fibrosis. Notably, the iron overload‐induced cellular senescence observed in OE mirrors the pattern of iron accumulation associated with age‐related ovarian aging, highlighting a potential convergence in the mechanisms underlying pathological and physiological ovarian decline. Nevertheless, OEI encompasses unique pathological features not typically observed in normal ovarian aging, such as persistent inflammation and immune cell infiltration in the follicular microenvironment. These inflammatory cues may amplify the detrimental effects of iron overload, creating a form of “inflammatory aging” that accelerates follicular depletion and impairs reproductive potential. Thus, while aged ovaries provide a valuable reference for understanding the long‐term impact of iron dysregulation, OEI represents a context‐specific model in which iron‐induced oxidative stress and inflammation converge to exacerbate ovarian aging. By highlighting both the convergent and divergent molecular features of OEI and ovarian aging, our study underscores the importance of iron homeostasis in maintaining ovarian health and offers new insights into how perturbations in iron metabolism may contribute to infertility across a range of clinical contexts.

This study has several limitations. First, due to ethical restrictions and limited clinical availability, we were unable to obtain ovarian tissue samples from OEI patients, which prevented direct examination of the ovarian microenvironment. Our findings are thus based on analyses of follicular fluid and in vitro granulosa cell models. Second, while our data demonstrate that iron overload induces senescence and mitochondrial dysfunction in granulosa cells, we did not directly assess its effects on oocyte quality. Future studies employing cumulus–oocyte complex COC models may provide a more accurate understanding of how iron overload influences oocyte competence.^[^ ^61^ ^]^ Last, although our findings suggest a potential therapeutic role for iron chelation, the clinical translation of this approach requires further in‐depth mechanistic studies and validation in preclinical models to assess safety, efficacy, and targeted delivery strategies.

In conclusion, our data highlight the potential of single‐cell sequencing and high‐resolution spatial transcriptomics to deepen our understanding of the pathological mechanisms underlying follicular development disorders in OEI. These approaches provide a rich dataset for further exploration of the complex intercellular interactions that contribute to reproductive dysfunction in the context of iron overload.

## 4 Experimental Section

### Retrospective IVF/ICSI Cohort Study Analysis

This retrospective cohort study included women who underwent IVF/ICSI‐FET treatment at the Reproductive Center of the Ninth People's Hospital affiliated with Shanghai Jiao Tong University School of Medicine from January 2010 to December 2018. The study population was divided into three groups: patients with ovarian endometriosis‐related infertility (OE group), male factor infertility (Male factor group), and tubal factor infertility (Tubal factor group). This study was approved by the Medical Ethics Committee of the First Affiliated Hospital of Naval Medical University (CHEC 2019–100) and conducted in accordance with the Helsinki Declaration. Informed consent was not required due to the retrospective nature of the study, and patient data were anonymized.

Inclusion criteria for the OE group were: (1) pathological confirmation of ovarian endometriosis; (2) regular sexual intercourse without contraception for at least 1 year without achieving natural pregnancy; (3) normal semen analysis of the male partner (according to Kruger criteria^[^ ^62^ ^]^); (4) female age between 25–40 years. Inclusion criteria for the Male factor group were: (1) cohabitation and regular sexual intercourse without contraception for at least 1 year, resulting in infertility due to male factor; (2) healthy female partner aged 25–40 years. Inclusion criteria for the Tubal factor group were: (1) diagnosis of tubal obstruction with infertility duration exceeding 1 year; (2) female age between 25 and 40 years; (3) normal semen analysis of the male partner.

Exclusion criteria for all groups included: ovulatory disorders such as polycystic ovary syndrome, adenomyosis, hyperthyroidism, systemic lupus erythematosus, or other autoimmune diseases; HIV infection or any active infection; smoking habits; use of hormonal or anti‐inflammatory medications within 3 months prior to treatment; any contraindications to ovarian stimulation; and incomplete historical data.

To address confounding factors and significant differences among the groups, propensity score matching (PSM)^[^ ^23^ ^]^ was employed. PSM balanced baseline clinical characteristics including couple's age, BMI, parity, duration of infertility, previous IVF/ICSI cycles, and FET endometrial preparation among the three groups. After PSM, a total of 924 patients (308 per group) were selected for further analysis. PSM was performed using the Matching package (v4.10)^[^ ^63^ ^]^ in R (v4.3.1).

### Patient Samples

All follicular fluid samples were obtained with written informed consent from participants, in accordance with the Declaration of Helsinki 2000 guidelines. Follicular fluid samples were collected from six patients undergoing IVF/ICSI at the Reproductive Center of the First Affiliated Hospital of Naval Medical University for single‐cell sequencing analysis. Three patients, classified as the OE group, underwent IVF due to ovarian endometriosis‐associated infertility. The other three patients, classified as the control group, underwent ICSI due to male factor infertility. Detailed baseline information of the 6 patients is provided in Table S9, Supporting Information. To assess iron ion levels, samples from 40 patients were analyzed. Twenty patients in the EMS group underwent IVF/ICSI due to endometriosis‐related infertility, while the control group included 20 patients treated for male factor, tubal factor, or non‐endometriosis‐related infertility.

Patients followed various ovarian stimulation protocols as determined by their clinicians. When a dominant follicle reached at least 18 mm in diameter, 0.25 mg of hCG was administered. Specifically, this work only aspirated follicles with a diameter >8 mm for downstream single‐cell analysis. These follicles are typically classified as antral follicles and are considered to be at a comparable maturation stage. By avoiding the inclusion of smaller or atretic follicles, this work aimed to minimize transcriptional variability arising from differing developmental stages. Oocyte retrieval was performed 34–38 h later under transvaginal ultrasound guidance. Retrieved oocytes were used for IVF/ICSI procedures, and follicular fluid was reserved for subsequent research. This study was approved by the Medical Ethics Committee of the First Affiliated Hospital of Naval Medical University (CHEC 2019–100).

### Follicular Fluid Sample Processing

After collecting and retrieving oocytes from IVF/ICSI superovulation patients, the oocyte‐cumulus complex was mechanically dissected post‐retrieval. This approach was chosen because most OEI patients in our cohort underwent conventional IVF rather than ICSI, due to female‐factor infertility, and thus did not require complete enzymatic denudation of oocytes. This work believed that avoiding enzymatic treatment helps preserve the native transcriptomic landscape of cumulus cells and minimizes gene expression artifacts that may be introduced by enzymatic exposure. The cumulus cell complexes were washed twice in DMEM/F12 medium (Gibco) containing 10% fetal bovine serum (FBS, Gibco) to remove MOPS solution and mineral oil residues from the oocyte collection process. Follicular fluid (excluding oocytes and cumulus cell complexes) was collected from patients undergoing IVF/ICSI. Red blood cell lysis buffer (YEASEN, 40401ES60) was added to the follicular fluid, and pipetting was performed to lyse the red blood cells. The mixture was then centrifuged. The resulting cell pellet was washed twice with PBS and resuspended in DMEM/F12 medium containing 10% FBS. The suspension was then combined with cumulus cells, stored at 4 °C, and subsequently used for single‐cell RNA sequencing and Bulk RNA sequencing.

### Iron Ion Assessment

Follicular fluid collected from patients undergoing ovarian stimulation for IVF/ICSI was centrifuged. The supernatant was used for iron ion analysis. Iron levels were measured using an iron assay kit (A039‐1‐1, Nanjing Jiancheng, China) according to the manufacturer's instructions.

### RNA Extraction, Bulk RNA‐seq Library Construction

Total RNA was isolated from samples utilizing the TRIzol reagent (Invitrogen, USA). The quality of isolated RNA was examined by NanoDrop ND‐1000 (Nano­Drop, DE, USA) and Agilent 2100 Bioanalyzer (Agilent, Santa Clara, CA). The qualified samples were then subjected to paired‐end library preparation according to the Illumina protocol (Illumina, San Diego, CA). The libraries were sequenced to produce paired‐end reads on an Illumina NovaSeq sequencing platform.

### Bulk RNA‐seq and Smart‐seq2 Single Cell Analysis

The resulting cell pellet was washed twice with PBS and RNA was extracted from cells using TRIzol reagent (Invitrogen) for RNA‐seq. Single cell data of oocyte cells were obtained from PRJNA514416,^[^ ^24^ ^]^ while external bulk data of granulosa cells were sourced from PRJNA806789. Raw sequencing data were processed using Trimmomatic software (v.0.3.6)^[^ ^64^ ^]^ to generate clean reads, which were then mapped to the GRCh38 reference genome using Hisat2 (v.2.2.0).^[^ ^65^ ^]^ Expression count matrices were generated using FeatureCounts (v.2.0.3).^[^ ^66^ ^]^ Differential expression analysis and enrichment analysis between different sample groups was performed using DESeq2 (v.1.40.2)^[^ ^67^ ^]^ and clusterProfiler (v.4.8.3).^[^ ^68^ ^]^

### BD Rhapsody Library Preparation and Sequencing

Single‐cell RNA sequencing was performed by NovelBio Bio‐Pharm Technology Co., Ltd. using the BD Rhapsody system. Library preparation followed the manufacturer's protocol. Single‐cell capture was achieved by randomly distributing single‐cell suspensions into over 20 000 microwells using limited dilution. Barcoded beads were added to pair with the cells in the microwells. Cells were lysed, allowing mRNA to hybridize with the barcoded oligonucleotides on the beads. The beads were collected for reverse transcription and ExoI digestion. Each cDNA molecule was tagged at the 5′ end with a unique molecular identifier (UMI) and a cell barcode. Whole transcriptome libraries were prepared using the BD Rhapsody Single‐Cell Whole Transcriptome Amplification (WTA) workflow, including random priming and extension (RPE), RPE amplification PCR, and WTA indexing PCR. Libraries were quantified using the High Sensitivity DNA chip on a Bioanalyzer 2200 (Agilent) and the Qubit High Sensitivity DNA Assay (Thermo Fisher Scientific). Sequencing was performed on Novaseq 6000 system (Illumina, CA, USA) with 150 bp paired‐end reads. For each scRNA‐seq library, reads were aligned, quantified, and subjected to initial quality control using the BD Genomics Rhapsody Analysis pipeline (v 2.2.1) with the RhapRef Human WTA reference genome (bd‐rhapsody‐public.s3‐website‐us‐east‐1.amazonaws.com/Rhapsody‐WTA/), following default parameters. The resulting filtered cell count matrices were used for downstream analysis.

### Downstream scRNA‐seq Analysis—scRNA‐seq Data Decontamination

Environmental RNA contamination in each single‐cell sample matrix was removed using the decontX package (v1.0.0).^[^ ^69^ ^]^ The *decontX* function in R (v4.3.1) assessed the contamination index for each cell. Cells with contamination scores exceeding 0.2 were excluded, following literature recommendations.

### Doublet Detection and Removal

Doublets were identified and removed using DoubletFinder (v.2.0.3),^[^ ^70^ ^]^ an algorithm designed to predict doublets in scRNA‐seq data. The expected doublet rate for each sample was set to 0.05. Cells identified as doublets were subsequently excluded from each sample.

### Quality Filters, Batch Effect Correction and Clustering

Basic quality control was performed on the samples. Genes expressed in fewer than 3 cells and cells expressing fewer than 200 genes or with mitochondrial content over 35% were excluded. Seurat (v4.1.0)^[^ ^71^ ^]^ was used to normalize the gene‐cell matrix and identify highly variable genes (HVGs) for unsupervised clustering. Principal component analysis (PCA) was performed on the top 2000 HVGs. The number of significant principal components (PCs) was determined using an elbow plot generated by *ElbowPlot* function. Batch effect correction was conducted using Harmony (v.0.1.1)^[^ ^72^ ^]^ with default parameters to remove batch effects in the PCA space before clustering analysis or cell type identification. Harmony's effectiveness in integrating batches while maintaining cell type separation was carefully evaluated. *FindNeighbors* function was used to construct a shared nearest neighbor (SNN) graph for unsupervised clustering using the *FindClusters* function. Different resolution parameters were tested to determine the optimal number of clusters with distinct transcriptional profiles, based on cluster marker genes. For visualization, dimensionality reduction was further performed using the uniform manifold approximation and projection (UMAP) method with *RunUMAP* function.

### Differential Expression Gene Analysis

DEGs for each cluster were identified using the *FindAllMarkers* function in the Seurat R package. DEGs were filtered based on the following criteria: expression in at least 10% of cells in the more abundant group, Log_2_(fold change) >0.25, and *P* value <0.05. After annotating the cell types for each cluster, the *FindMarkers* function was used to identify DEGs between OE and CON groups within each cell type.

### Annotation of scRNA‐seq Datasets

Bubble plots were generated based on marker genes for cell types in follicular fluid as previously reported. Each cluster was assigned a cell type label based on the expression of marker genes and DEGs, including granulosa cells, stromal cells, and immune cells (T cells, NKT cells, B cells, DCs, and monocytes/macrophages). Granulosa cells were divided into three spatially distinct clusters during unsupervised clustering, prompting further investigation of their functions. GO term enrichment analysis was performed on the top 50 DEGs for each cluster (see Enrichment Analysis Methods). Each cluster's function was defined based on the enriched terms.

### Enrichment Analysis

Enrichment analysis was conducted using the clusterProfiler package. GO and KEGG enrichment analyses were performed with the *enrichGO* and *enrichKEGG* functions, respectively. Redundant GO terms were removed using the simplify function with the parameter “cutoff = 0.5.” GO terms with a *p* value <0.05 were considered significantly enriched. GSEA was performed using the *gseGO* and *gseKEGG* functions.

### SCENIC Analysis

SCENIC (Single‐Cell Regulatory Network Inference and Clustering)^[^ ^30^ ^]^ was used to predict transcription factor (TF) co‐expression. SCENIC utilized the count matrix of all cells. After filtering based on default expression parameters, GRNBoost in pySCENIC (v.0.10.4) was used to infer potential TF targets across all cells. The resulting co‐expression modules were integrated with the cisTarget database to identify putative regulators. Finally, the activity of 347 validated regulators was scored in each cell. To identify TFs with increased activity in specific cell types and between groups, Wilcoxon Rank Sum test from Seurat was applied onto the z‐transformed “cell × TF” activity matrix in a one‐versus‐all fashion.

### Quantification of Sample Enrichment

To quantify sample enrichment for each cell subpopulation in follicular fluid, this work calculated the ratio of observed to expected cell numbers (Ro/e) for each cluster across different samples, as described in previous studies.^[^ ^73^ ^]^ For a given cell cluster, Ro/e > 1 indicates that cells of this cluster are more frequently observed in a specific sample than expected by chance (enrichment), while Ro/e < 1 indicates that cells are observed less frequently than expected (depletion).

### Gene Set Score Analysis

Gene set score analysis was performed using the *AddModuleScore* function in Seurat to calculate module scores of gene expression in scRNA‐seq. Gene sets were obtained from the KEGG^[^ ^74^ ^]^ and MSigDB^[^ ^75^ ^]^ databases. Additionally, the iron‐homeostasis pathway map was created based on the KEGG database (hsa04216) and relevant literature.^[^ ^76^ ^]^

### Data Imputation

To enhance the representation of gene correlations, data imputation was performed using MAGIC (v.2.0.3)^[^ ^77^ ^]^ to further estimate and smooth the normalized count matrix. Visualization of the results was done using ggplot2 (v.3.4.3).

### Cell‐Cell Communication Analysis

Cell‐cell interactions were evaluated using CellChat (v.1.6.1).^[^ ^78^ ^]^ This work followed the default pipeline, analyzing cell‐cell interactions separately for different groups. Normalized count data for each condition were used to create CellChat objects, and recommended preprocessing functions were applied to analyze each dataset using default parameters. CellChatDB.human was employed as the reference database for inferring cell‐cell communication, utilizing all categories of ligand‐receptor interactions available. Interactions involving fewer than 10 cells were excluded from the analysis. Additionally, stromal cells were excluded due to their low abundance (<0.1%), which could bias the communication analysis. The results generated by CellChat were visualized using ggplot2.

### Single‐Cell Trajectory Inference

To investigate the differentiation trajectories of the Granulosa1 cell subpopulation, the Monocle2 package (v.2.8.3)^[^ ^79^ ^]^ was employed with recommended default parameters. First, the gene expression matrix for specific cell types was exported from Seurat to Monocle to construct a cell dataset. The *setOrderingFilter* function was applied to order cells based on highly variable genes (q < 0.001). Dimensionality reduction was then performed using the *reduceDimension* function with the DDRTree method. The differentiation process was visualized using the *plot_pseudotime_heatmap* function to reveal a series of representative key genes. The *plot_cell_trajectory* function was used to plot the trajectory of each group along the same pseudotime axis. The BEAM results were exported as plotting objects using the *plot_genes_branched_heatmap2* function from the ClusterGVis package (v0.1.1).

### Metabolic Flux Analysis

Metabolic flux was calculated using scFEA (v1.1.2)^[^ ^41^ ^]^ and scMetabolism (v.0.2.1).^[^ ^42^ ^]^ The raw count matrix of the Granulosa1 cell subpopulation was used as input for scFEA.py script, with recommended parameters to determine the metabolic flux for each cell. For scMetabolism, the Seurat object containing all cells was utilized, with the method parameter set to “VISION” to calculate metabolic activity for each cell. The metabolic effect size between different groups was subsequently calculated using the *cohens’ D* function from the lsr package (v 0.5.2).

### Mfuzz Analysis

The normalized single‐cell gene expression data were aggregated by cell type and age group to create a pseudo‐bulk gene expression matrix. Following the tutorial's guidelines, genes were filtered prior to clustering, and the remaining genes' normalized data were subjected to Z‐score transformation. Clustering was then performed using the c‐means fuzzy clustering method, resulting in distinct clusters (v.2.60.0).^[^ ^80^ ^]^

### Animals

All animal experiments were carried out at the Central Laboratory of Changhai Hospital and approved by the Experimental Animal Ethics Review Committee (Registration no. CHEC(AE) 2022‐002). The iron overload mouse model was established with slight modifications based on previously reported methods.^[^ ^11^ ^]^ Female Kunming mice (Vital River, China) were 7‐week‐old on the day of arrival. After a 5‐day acclimatization period, the mice in the iron overload group received intraperitoneal injections of dextran iron solution (0.5 g kg^−1^) every 3 days, while control mice received equivalent volumes of saline. After 3 weeks, to minimize variability due to the estrus cycle and to better simulate the pre‐ovulatory state of IVF patients, all mice were intraperitoneally injected with pregnant mare serum gonadotropin (PMSG) 48 h prior to sample collection. Fresh ovarian tissues were washed three times with PBS, rapidly frozen in liquid nitrogen within Tissue‐Tek OCT (Sakura, 4583), and stored at −80 °C.

### Stereo‐seq Library Construction

Stereo‐seq library construction was performed by Annoroad Gene Technology. ≈10 mm frozen sections were attached to a Stereo‐seq capture chip (1 cm × 1 cm) with a spot size of 220 nm and a center‐to‐center distance of 715 nm, and incubated at 37 °C for 6 min. The tissue slides were fixed and stained with nucleic acid dyes to assess post‐sequencing resolution. Adjacent sections were subjected to H&E staining for histological examination. The attached tissue sections were permeabilized to release RNA, which was then captured by DNA nanoballs (DNBs) on the chip. The captured RNA was reverse transcribed into cDNA in situ at 42 °C. Following tissue digestion and exonuclease I treatment, cDNA was amplified. For library construction, 20 ng of cDNA was fragmented and amplified. Purified PCR products were used for DNB generation and sequenced on an MGI DNBSEQ‐Tx sequencer (MGI, China).

### Stereo‐seq Raw Data Processing

Read 1 sequences of Stereo‐seq data containing coordinate identity (CID, 1–25 bp) and UMI (26–35 bp) were mapped to the CID, and corresponding read 2 cDNA sequences were aligned to the mm10 (Ensembl 93) mouse reference genome using STAR (v.2.7.9a).^[^ ^81^ ^]^ Mapped reads were filtered with MAPQ ≥ 10 and annotated. UMIs with identical CIDs mapping to the same gene were aggregated to generate a count matrix, including gene ID, X coordinate, Y coordinate, and UMI count. This process was integrated into an internal pipeline available on GitHub (SAW; https://github.com/BGIResearch/SAW). The count matrix was binned into 50 (50 × 50 DNA nanoballs, each with a center‐to‐center distance of 715 nm; each bin with a resolution of 36 × 36 µm) for downstream analysis.

### Downstream Stereo‐seq Analysis—Quality filters

Basic quality control was performed on the samples. Each bin50 genes < 1000 or with mitochondrial content over 5% were excluded.

### Cell Type Deconvolution

To deconvolute cell type composition within each Stereo‐seq bin, this work employed RCTD (v.1.2.0).^[^ ^82^ ^]^ The *Reference* function was used to generate a reference object based on the expression matrix and cell type information from mouse ovarian tissue.^[^ ^22^ ^]^ Expression matrices and coordinate information from each steady‐state section were exported to construct a query object using the *SpatialRNA* function. The final deconvolution results were obtained using the core functions *create_RCTD* and *run_RCTD*. The final distribution of each cell type was determined by the average cell proportion weight in each Stereo‐seq bin50.

### Gene Set Score Analysis

The top 50 marker genes of Granulosa1, Granulosa2, and Granulosa3 from human follicular fluid single‐cell data were converted to mouse homologs using biomaRt (v.2.56.1).^[^ ^83^ ^]^ The *AddModuleScore* function was then used to compute the spatial distribution of these genes in the Stereo‐seq data, visualized using the *FeaturePlot* function. Mouse gene sets were obtained from KEGG and MSigDB and visualized using the same method. Additionally, aging‐related gene sets were sourced from the Aging Atlas.^[^ ^47^ ^]^ These were intersected with upregulated and downregulated DEGs from the Aging Atlas to derive gene sets associated with aging.

### SASP Assay

The levels of SASP factors, including MCP‐1, IL‐8, IL‐1β, IL‐6, and GM‐CSF, in individual follicular fluid samples were measured using specific enzyme‐linked immunosorbent assays (ELISA) according to the manufacturer's instructions (JONLNBIO, China).

### Cell Culture

KGN cells were cultured in DMEM/F12 medium (Gibco) supplemented with 10% fetal bovine serum (FBS, Gibco) and 1% penicillin/streptomycin (Gibco) at 37 °C in a humidified incubator with 5% CO₂ for 18 h. To mimic the follicular fluid (FF) microenvironment, cells were treated with 20% FF derived from different patients for indicated experiments. For in vitro iron overload modeling, KGN cells were seeded in 6‐well plates at a density of 1  ×  10⁶ cells per well and treated with 1.5 mM ferric ammonium citrate (FAC, sigma) to induce iron accumulation. In selected experiments, 1 mM deferoxamine mesylate (DFO, sigma) was co‐administered with FAC to evaluate the protective effects of iron chelation.

### SA‐β‐Gal Assay

SA‐β‐gal assay was carried out according to the manufacturer's instruction (40754ES60, YESEN). Treated KGN were seeded on 12‐well plates at a density of 5 × 10^5^ cells mL^−1^. After 24 h, cells were washed, fixed and stained overnight at 37 °C. Cells were imaged and photographed using a live‐cell imaging microscope.

### Mitochondrial Related Assays

Cells were stained with different dyes for confocal microscopy analysis. To measure mitochondrial membrane potential, cells were stained with 2 µM JC‐1 (M34152, Invitrogen). Mitochondrial ROS was measured by incubating cells with 500 nM MitoSOX Red (M36008, Invitrogen) and with Hoechst 33342 to visualize nuclear. To measure the fluorescence intensity of JC‐1 and MitoSOX Red, ImageJ software was employed.

### Immunofluorescence Staining and Imaging

Paraffin‐embedded sections (5 µm thick) were deparaffinized in xylene and antigen retrieval was performed in EDTA antigen retrieval buffer (pH 8.0) at 100 °C for 27 min. Sections were then washed three times with PBS for 5 min each. Subsequently, they were blocked with 3% BSA or for 30 min. Primary antibodies were applied and sections were incubated overnight at 4 °C. The next day, sections were washed three times with PBS and then incubated with appropriate secondary antibodies at 37 °C in the dark for 1 h. Finally, sections were counterstained with 4,6‐diamidino‐2‐phenylindole (DAPI) for 10 min in the dark. After washing, sections were mounted with antifade mounting medium for immunofluorescence. Images were acquired using a digital scanner (Pannoramic MIDI, 3DHISTECH) under different fluorescence channels. ImageJ software was used to measure average fluorescence intensity or area. Antibodies used for immunofluorescence are listed in Table S20, Supporting Information.

### Statistical Analyses

Statistical analyses were conducted using R (v.4.3.1). Bar graphs were presented as mean ± s.e.m. (standard error of the mean). Details regarding the calculation of *p* values and the number of replicates for each experiment are provided in the figure legends. Box plots depict the median (center line), interquartile range (box), and whiskers extending to 1.5 times the interquartile range.

### Ethical Statement

The patient ethics was approved by the Medical Ethics Committee of the First Affiliated Hospital of Naval Medical University (CHEC 2019–100). All animal experiments were approved by the Experimental Animal Ethics Review Committee (Registration no. CHEC(AE) 2022‐002).

## Conflict of Interest

The authors declare no conflict of interest.

## Author Contributions

Y.L., W.Z., J.D., and D.S. contributed equally to this work. Y.G., C.Y., and Z.N. conceived the study and supervised the overall experiments. Y.L., J.D., and W.Z. planned and designed the experiments. D.S. performed isolation of follicular fluid. Y.L. processed the raw data and performed computational analyses. S.S and X.L. performed ovary histological analysis. Q.Z. created the final figures and illustrations. S.M. organized and uploaded codes and dataset. Y.L. and Z.N. drafted the manuscript with input from C.Y., M.L and Y.G. M.L., W.C. and J.Y. performed manuscript review and editing. All authors read and approved the manuscript.

## Supporting information



Supporting Information

Supporting Tables

Supporting Tables

## Data Availability

The bulk RNA‐seq data generated in this study have been deposited to the GEO (GSE276193). The raw data of scRNA‐seq and Stereo‐seq data have been deposited to CNGBdb under accession number CNP0006183 and CNP0006170. The full processed file of Smart‐seq2 data from PRJNA514416 and scRNA‐seq and Stereo‐seq data from our study has been easily accessible on Figshare (https://figshare.com/s/8f61bbe2395ae0df0e63).
