# Supplementary material for: Integrative Single‐Cell Analysis Reveals Iron Overload‐Induced Senescence and Metabolic Reprogramming in Ovarian Endometriosis‐Associated Infertility
Source: Adv Sci (Weinh). 2025 Jul 22;12(29):e17528. doi: 10.1002/advs.202417528 (PMC12362736; doi:10.1002/advs.202417528)
Supplement: Supplementary file 1 — Supporting Information [file ADVS-12-e17528-s003.pdf]

ADVANCED SCIENCE
Open Access

Supporting Information

for *Adv. Sci.*, DOI 10.1002/advs.202417528

Integrative Single-Cell Analysis Reveals Iron Overload-Induced Senescence and Metabolic Reprogramming in Ovarian Endometriosis-Associated Infertility

Yangshuo Li, Wei Zhou, Jie Ding, Di Song, Wen Cheng, Jin Yu, Shuai Sun, Shanshan Mei, Xiaolan Liang, Qianqian Zhao, Yanping Kuang, Mingqing Li, Zhexin Ni*, Chaoqin Yu* and Yue Gao**

Integrative Single-Cell Analysis Reveals Iron Overload-Induced Senescence and Metabolic Reprogramming in Ovarian Endometriosis-Associated Infertility

*Yangshuo Li, Wei Zhou, Jie Ding, Di Song, Wen Cheng, Jin Yu, Shuai Sun, Shanshan Mei, Xiaolan Liang, Qianqian Zhao, Yanping Kuang, Mingqing Li *, Zhexin Ni *, Chaoqin Yu *, Yue Gao**

Supporting Information

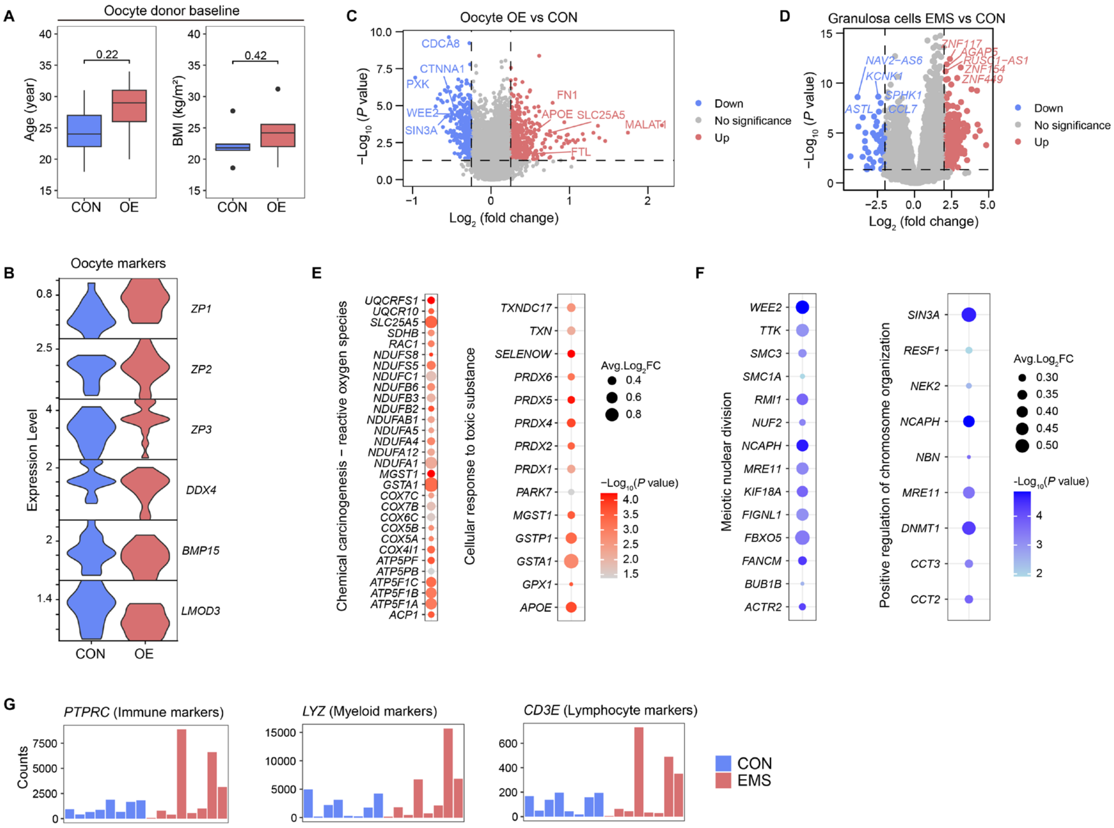

Figure S1 Transcriptomic differences in oocytes and granulosa cells between OEI patients and controls.

(A) Age and Body Mass Index (BMI) of ovarian endometriosis (OE) and healthy donors (CON) groups from oocyte Smart-seq2 single-cell RNA sequencing data. Two-tailed Student's *t*-test.

(B) Violin plot showing the expression levels of oocyte marker genes between the OE and CON groups.

(C) Volcano plot of differentially expressed genes (DEGs, *P* value < 0.05 and |Log2(Fold change)| > 0.25) in oocytes from the OE and CON groups.

(D) Volcano plot of DEGs (*P* value < 0.05 and |Log2(Fold change)| > 2) in granulosa cells from the endometriosis (EMS) and non-endometriosis (CON) groups.

(E-F) Upregulated (**E**) or downregulated (**F**) genes (|Log2(Fold change)| > 0.25) in GSEA of oocytes from the OE and CON groups. Corresponding to Figure 1I.

(G) Counts of immune cell marker genes in granulosa cell transcriptomic data.

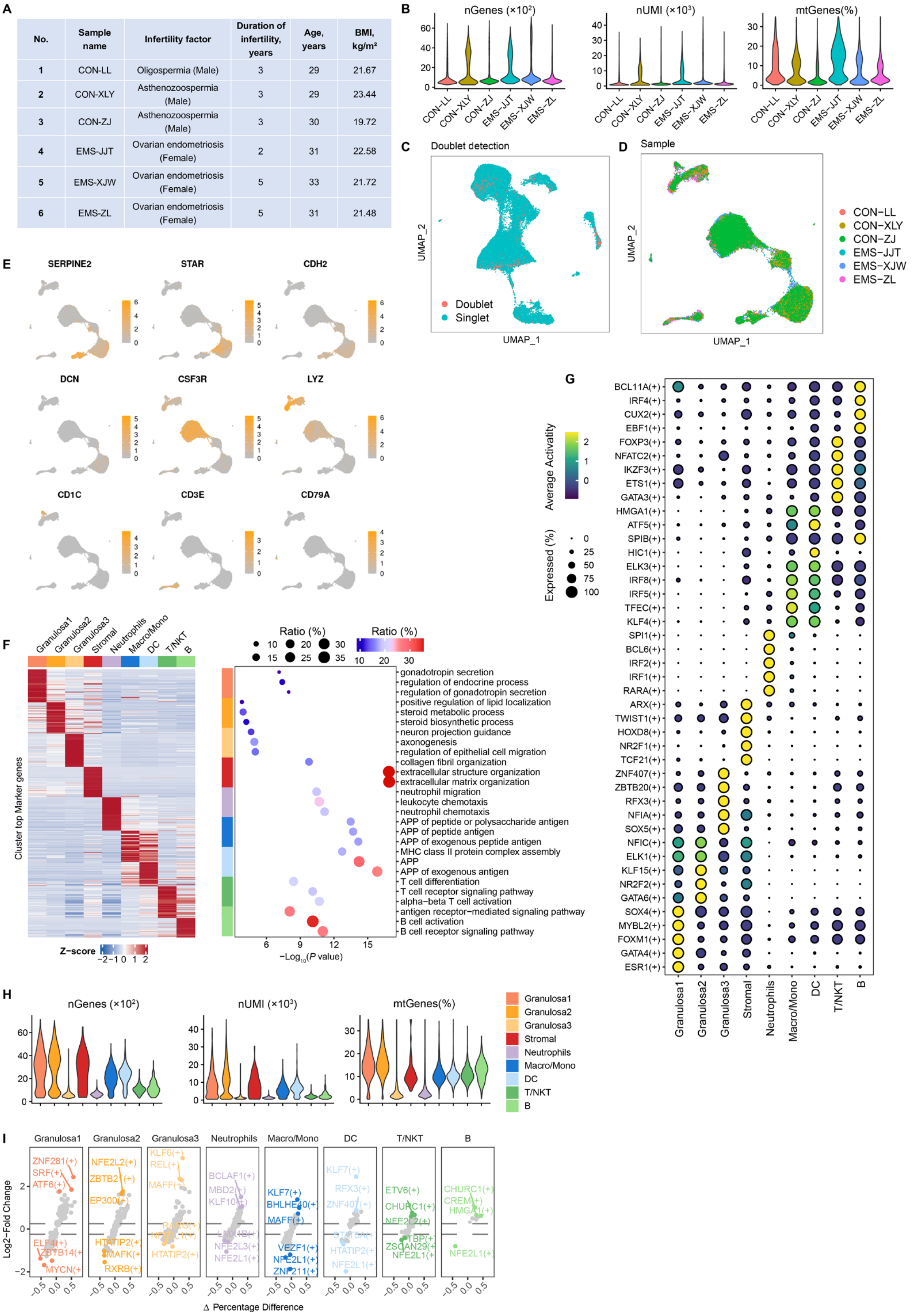

Figure S2 Quality control metrics and cell annotation procedures.

(A) Summary of patient information analyzed in this study.

(B) Number of reads, detected genes, and percentage of mitochondrial gene content after filtering in scRNA-seq data across different samples.

(C) UMAP visualization showing predicted doublets in the scRNA-seq data.

(D) UMAP visualization displaying the origin of major cell types across different samples.
(E) UMAP visualization of the expression of typical marker genes for major cell types.
(F) Scaled Z-scores of the top 50 specifically expressed genes for each cell type (left), and representative GO terms for these specifically expressed genes (right).
(G) Top 5 transcription factors (TFs) active in each cell subtype.
(H) Number of reads, detected genes, and percentage of mitochondrial gene content after filtering for different cell types in the scRNA-seq data.
(I) Differential transcription factor activity between OE and CON groups across cell types. Differential TF activity was defined by $|Log_2(\text{Fold change})| > 0.25$ and adjusted *P* value < 0.05. TFs upregulated in the OE group are shown above the dashed line, and those downregulated in the OE group are shown below. The top 3 upregulated and downregulated TFs for each cell type are highlighted.

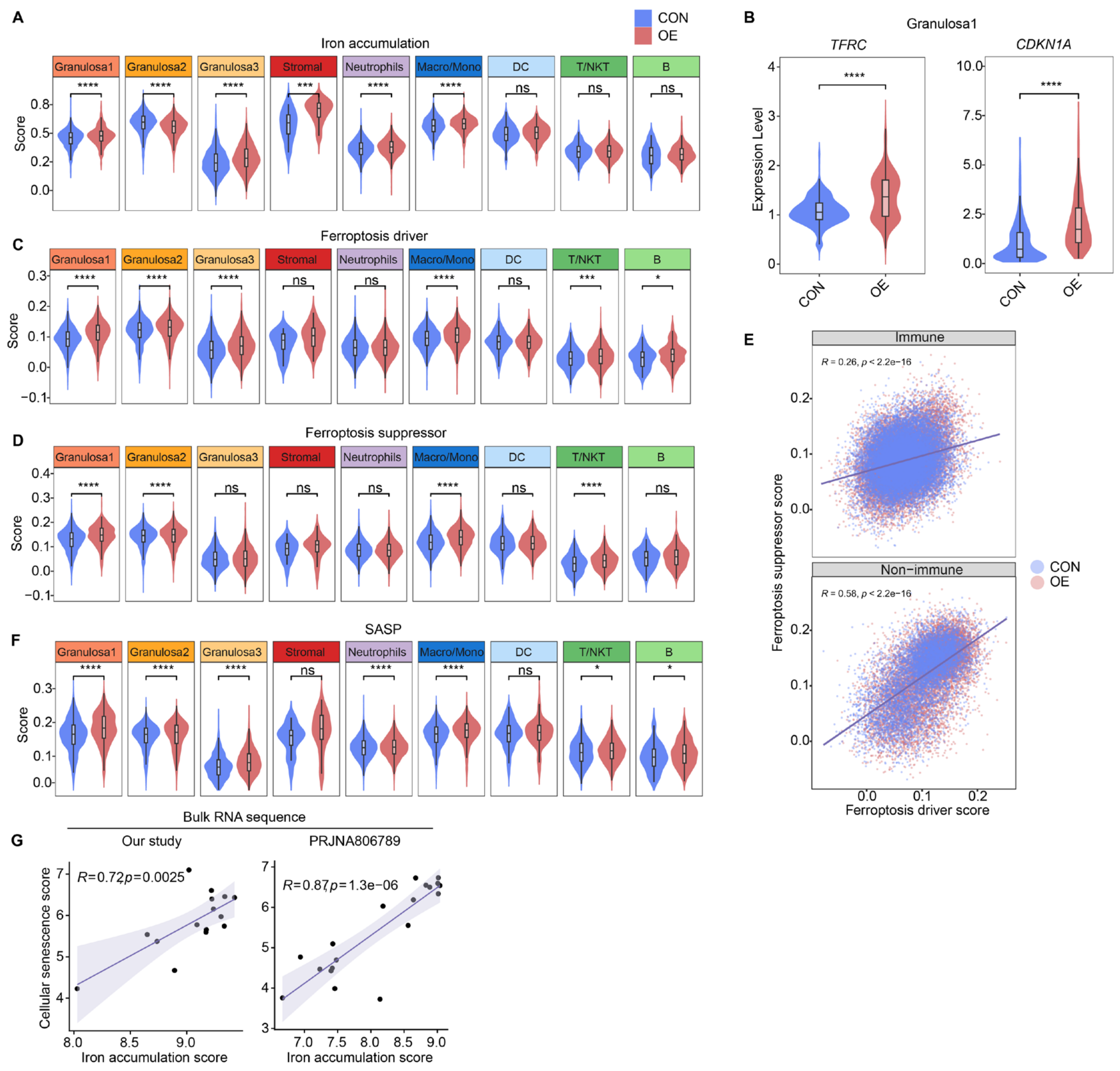

Figure S3 Response of different cell types to ferroptosis and cellular senescence-related gene sets in iron-overloaded follicular fluid.

(A, C, D, F) Gene set scoring analysis of iron accumulation (**A**), ferroptosis drivers (**C**), ferroptosis inhibitors (**D**), and SASP (**F**) in different cell types from follicular fluid scRNA-seq data of OE and CON groups.

(B) Expression levels of *TFRC* and *CDKN1A* genes in the Granulosa1 cluster between the OE and CON groups. Data in (**A-D, F)** were analyzed using the two-sided Wilcoxon rank-sum test. ****$P < 0.0001$; ***$P < 0.001$; *$P < 0.05$; ns, no significance.

(E) Pearson correlation between ferroptosis driver and inhibitor gene scores in immune and non-immune cells from follicular fluid scRNA-seq data.

(G) Pearson correlation between iron accumulation scores and cellular senescence scores in bulk RNA-seq data from follicular fluid.

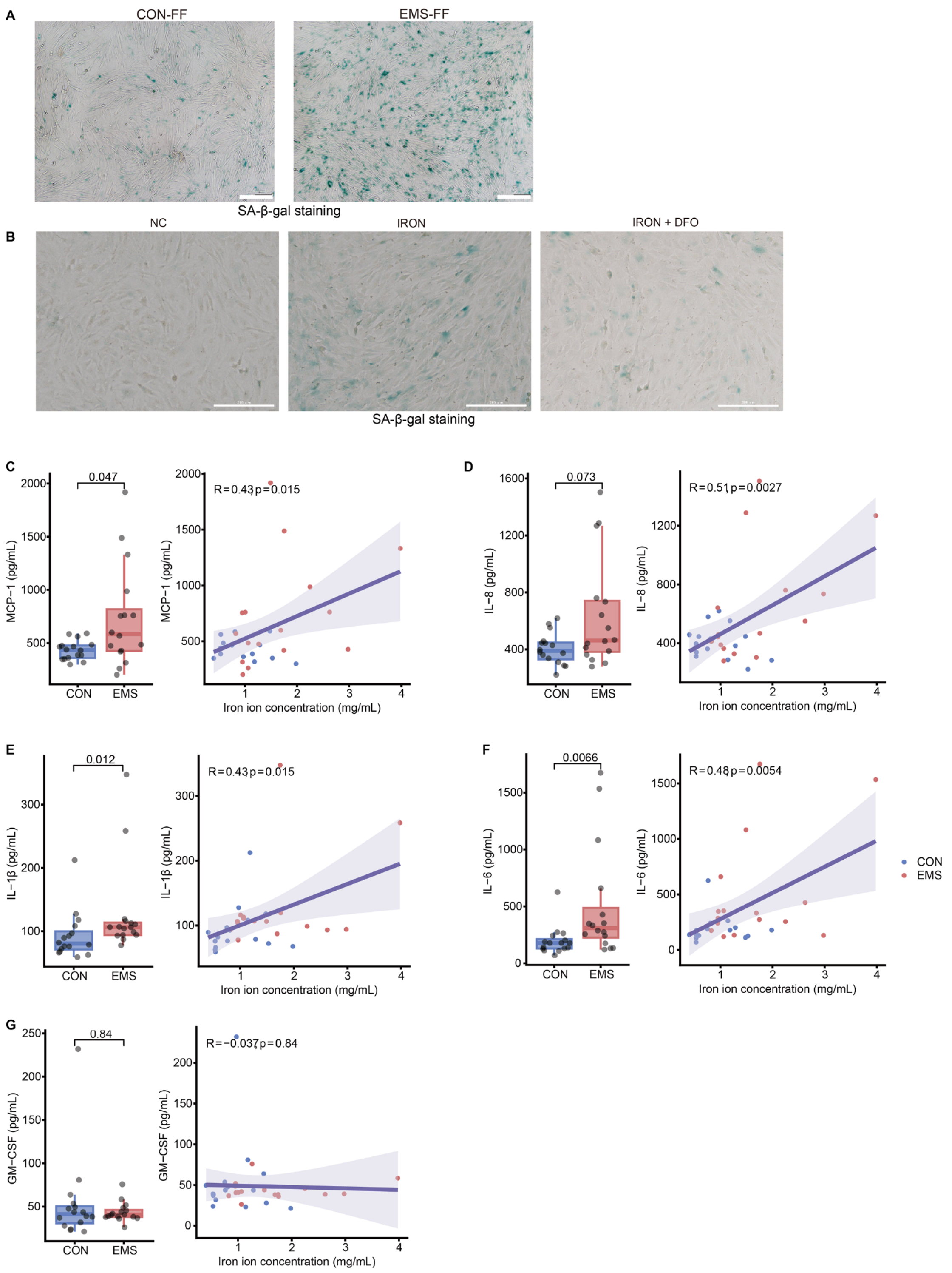

Figure S4 Association between iron overload and senescence features in the follicular fluid of patients with endometriosis-associated infertility.

(A) Granulosa cell line KGN cells were cultured with follicular fluid from control (CON-FF) or endometriosis (EMS-FF) patients. The experiments were repeated three times, and the representative images were presented. Scale bars, 200 μm.

(B) KGN cells were treated with Ammonium Ferric Citrate (IRON), with or without the iron chelator deferoxamine (IRON + DFO), and compared to the negative control (NC). Experiments were performed in triplicate, and representative images are shown. Scale bars, 200 μm.

(C, D, E, F, G) The senescence-associated secretory phenotype (SASP) factors MCP-1, IL-8, IL-1β, IL-6, and GM-CSF levels in follicular fluid of patients with endometriosis (EMS) and non-endometriosis (CON), left. Two-sided Wilcoxon rank-sum test. Pearson correlation analysis between iron ion levels in follicular fluid and MCP-1, IL-8, IL-1β, IL-6, and GM-CSF levels in corresponding patients, right. Points are colored by different groups.

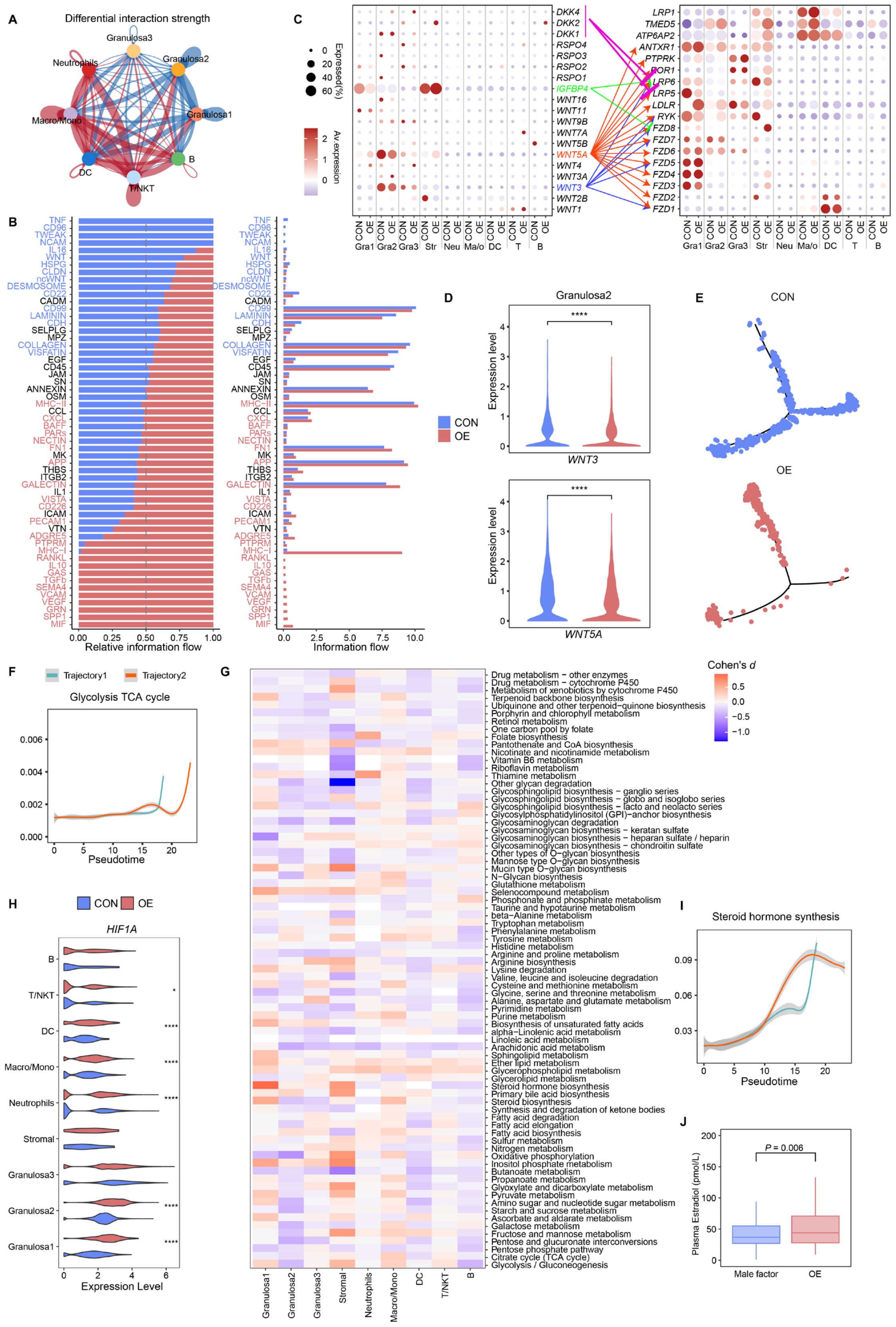

Figure S5 Cell-cell communication, monocle pseudotime, and metabolic flux analysis.

(A) Differential network of cell communication strength. Red (or blue) indicates increased (or decreased) signaling in the OE group compared to the CON group.

(B) Differences in overall information flow across all cell types between OE and CON groups.

Relative information flow (left) and absolute information flow (right). Red pathways are enriched in the OE group, while blue pathways are enriched in the CON group.

(C) Expression levels and percentages of inferred ligand-receptor pairs within the WNT pathway across all cell types. Arrow colors correspond to the pathways involved with the ligand-receptor pairs. Gra1, Granulosa1; Gra2, Granulosa2; Gra3, Granulosa3; Str, Stromal; Neu, Neutrophils; Ma/o, Macro/Mono; T, T/NKT.

(D) Expression levels of *WNT3* and *WNT5A* genes in the Granulosa2 cluster between the OE and CON groups. Two-sided Wilcoxon rank-sum test. ****$P < 0.0001$.

(E) Distribution of Granulosa1 cells along the Monocle-inferred trajectory in the CON and OE groups.

(F, I) Metabolic flux distribution along pseudotime in Trajectory 1 and Trajectory 2 for glycolysis TCA cycle (**F**) and steroid hormone biosynthesis (**I**) as inferred by scFEA.

(G) Heatmap displaying differences in metabolic flux between OE and CON groups across all cell types as predicted by scMetabolism. Red indicates enrichment in the OE group, while blue indicates enrichment in the CON group.

(H) Comparison of *HIF1A* gene expression levels across cell types between the groups. Two-sided Wilcoxon rank-sum test. *$P < 0.05$.

(J) Serum estrogen levels across different patient groups in the cohort analysis. Two-sided Wilcoxon rank-sum test.

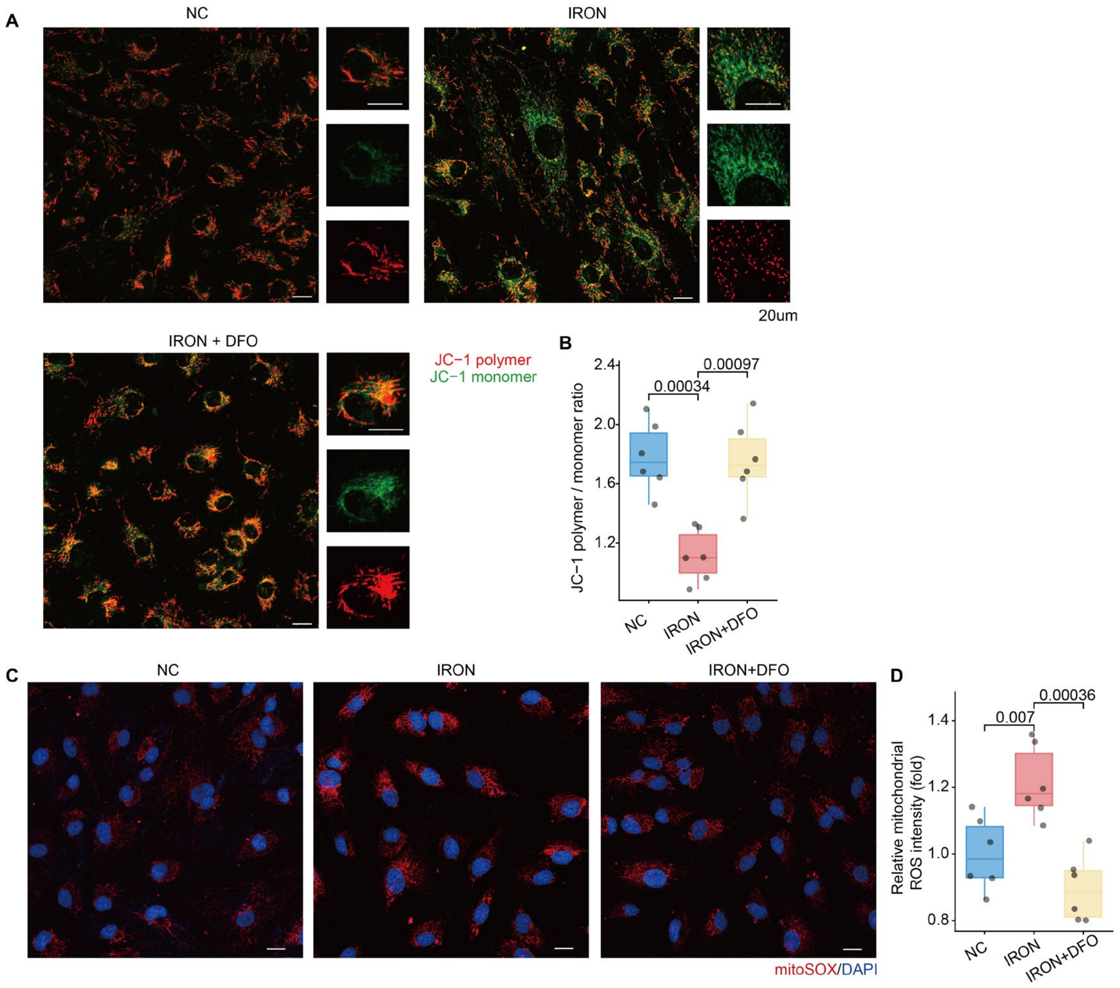

Figure S6 Excess iron impairs mitochondrial function in KGN cells.

(A, B) Mitochondrial membrane potential was assessed using JC-1 staining in KGN cells treated with Ammonium Ferric Citrate (IRON), with or without the iron chelator deferoxamine (IRON + DFO) (A). Scale bar, 20 μm. The ratio of JC-1 polymer to JC-1 monomer fluorescence intensity (B) was calculated. n = 6. Two-sided Wilcoxon rank-sum test.

(C, D) MitoSOX staining, reporting mitochondrial ROS levels (C). Scale bar, 20 μm. The relative mitochondrial ROS intensity was calculated (D). n = 6. Two-sided Wilcoxon rank-sum test.

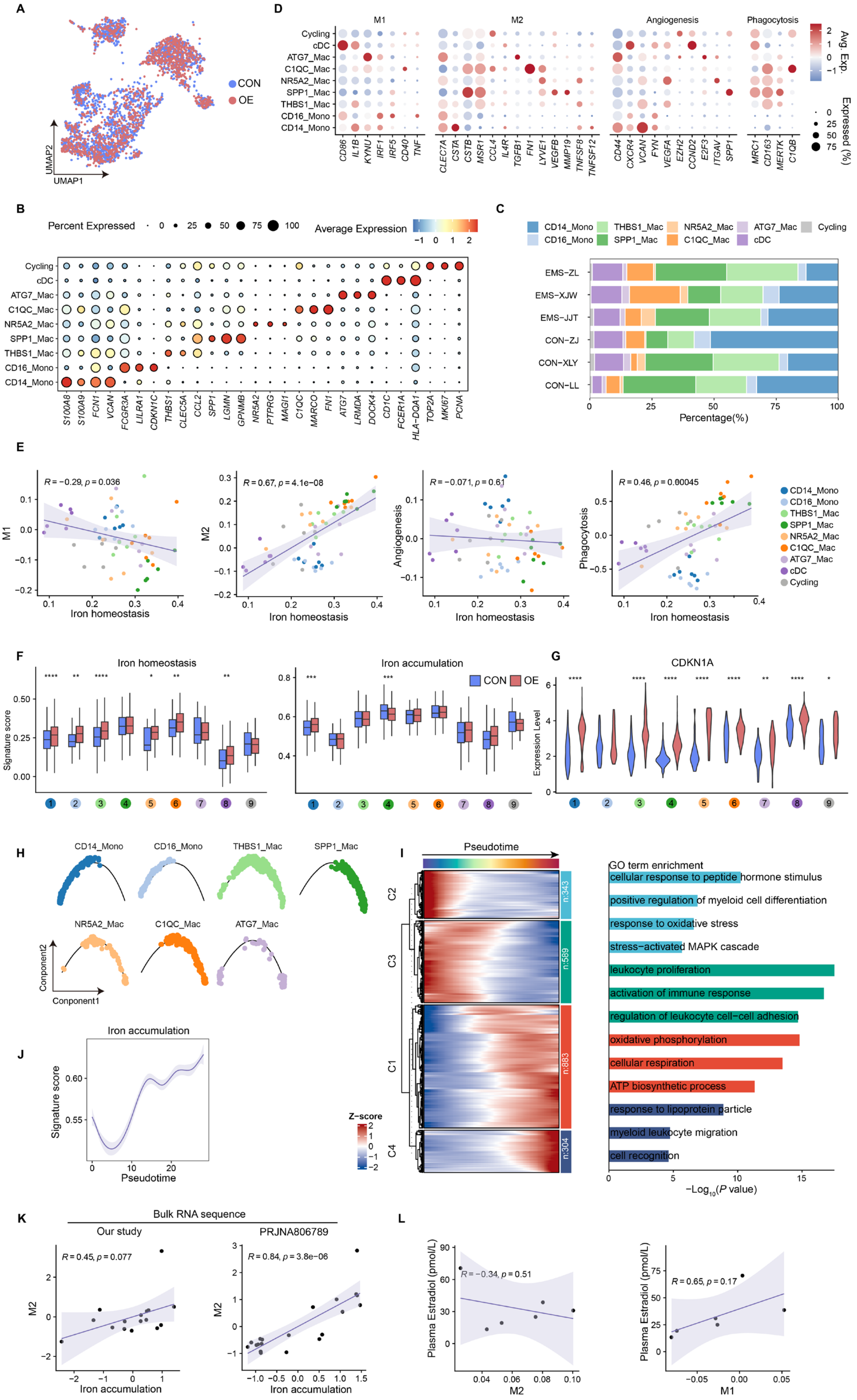
A
CON
OE
UMAP1
UMAP2
D
M1
M2
Angiogenesis
Phagocytosis
Avg. Exp.
Expressed (%)
B
Percent Expressed
Average Expression
C
Percentage(%)
EMS-ZL
EMS-XJW
EMS-JJT
CON-ZJ
CON-XLY
CON-LL
E
Iron homeostasis
F
Iron homeostasis
Iron accumulation
Signature score
G
CDKN1A
Expression Level
H
Conponent1
Conponent2
I
Pseudotime
GO term enrichment
cellular response to peptide hormone stimulus
positive regulation of myeloid cell differentiation
response to oxidative stress
stress−activated MAPK cascade
leukocyte proliferation
activation of immune response
regulation of leukocyte cell−cell adhesion
oxidative phosphorylation
cellular respiration
ATP biosynthetic process
response to lipoprotein particle
myeloid leukocyte migration
cell recognition
Z−score
J
Iron accumulation
Pseudotime
K
Bulk RNA sequence
Our study
PRJNA806789
Iron accumulation
L
Plasma Estradiol (pmol/L)

Figure S7 Phenotypic changes of M1/M2 in myeloid cells under iron accumulation conditions.

(A) UMAP visualization of myeloid cell clusters derived from follicular fluid across different groups.

(B) Dot plot showing the top gene expression levels and frequencies in each myeloid cell subcluster.

(C) Proportional composition of myeloid cell subclusters in each sample.

(D) Expression levels and frequencies of genes associated with M1-like, M2-like, angiogenic, and phagocytic within myeloid cell clusters.

(E) Pearson correlation between iron homeostasis features and M1-like, M2-like, angiogenic, and phagocytic gene expression in myeloid cell subclusters. Each dot represents a cell subcluster in different samples, color-coded by myeloid subcluster.

(F) Comparison of iron homeostasis (left) and iron accumulation (right) gene signatures between OE and CON groups across different myeloid cell clusters.

(G) Comparison of *CDKN1A* gene expression between OE and CON groups in different myeloid cell clusters. *P* values in (**F, G**) were calculated using the two-sided Wilcoxon rank-sum test. ****$P < 0.0001$; ***$P < 0.001$; **$P < 0.01$; *$P < 0.05$.

(H) Distribution of myeloid cell subclusters along Monocle-inferred trajectory.

(I) The gene expression along the developmental trajectory of Mono/Macro (left) and the enriched GO terms of clustered genes in (**H**). *P* values were calculated by hypergeometric test.

(J) Iron accumulation signature scores along the pseudotime axis.

(K) Pearson correlation between iron accumulation scores and M2 gene signatures in bulk RNA-seq data from follicular fluid.

(L) Pearson correlation between M2-like (left) and M1-like (right) gene signatures and serum estrogen levels.

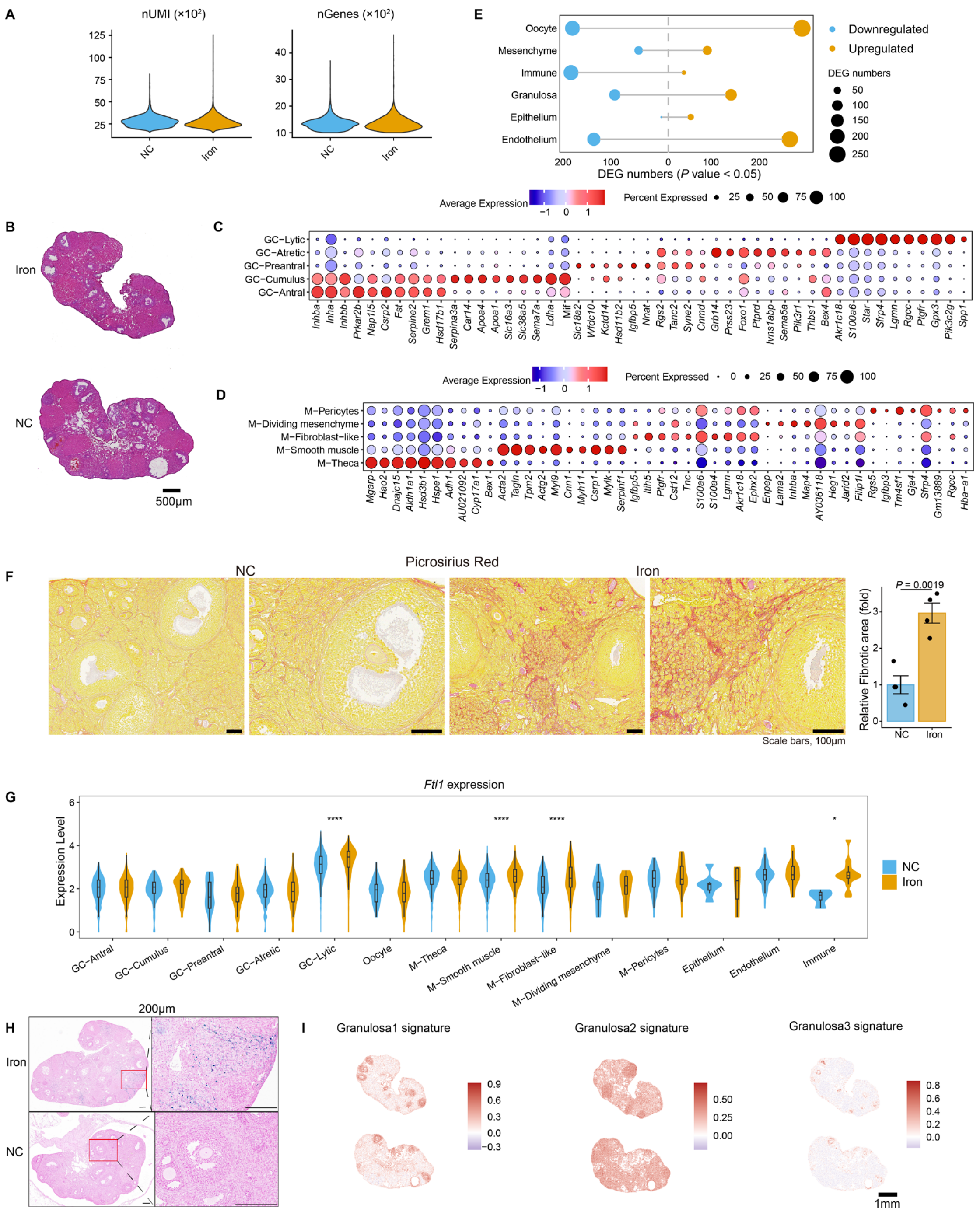

Figure S8. Spatial identification and visualization of ovarian cell types and characteristics in iron-overloaded mice using Stereo-seq, related to Figure 6.

(A) Summary of read counts, gene counts, and the percentage of mitochondrial genes after filtering in different samples from the Stereo-seq data.

(B) H&E staining of tissue sections adjacent to those used for Stereo-seq. Scale bars, 500μm.

(C-D) Dot plot showing the average expression and frequency of top marker genes for different granulosa (**C**) and mesenchymal (**D**) cell types in the ovary.

(E) Number of DEGs in the six major ovarian cell types between Iron and NC groups.

(F) Representative images of Picrosirius Red staining of ovaries in mice with iron overload (Iron) and negative control (NC). Two-sided Wilcoxon rank-sum test. Relative intensities are

quantified as fold changes and are represented as mean ± SEM. n = 4 for each group.

(G) Expression levels of the *Ftl1* gene across all cell types between the two groups. Two-sided Wilcoxon rank-sum test. ****P < 0.0001; *P < 0.05.

(H) Prussian blue staining of iron-overloaded mouse ovaries. Scale bars, 200μm.

(I) Spatial distribution of granulosa cell subclusters identified in follicular fluid scRNA-seq data, mapped onto the Stereo-seq spatial transcriptomics.

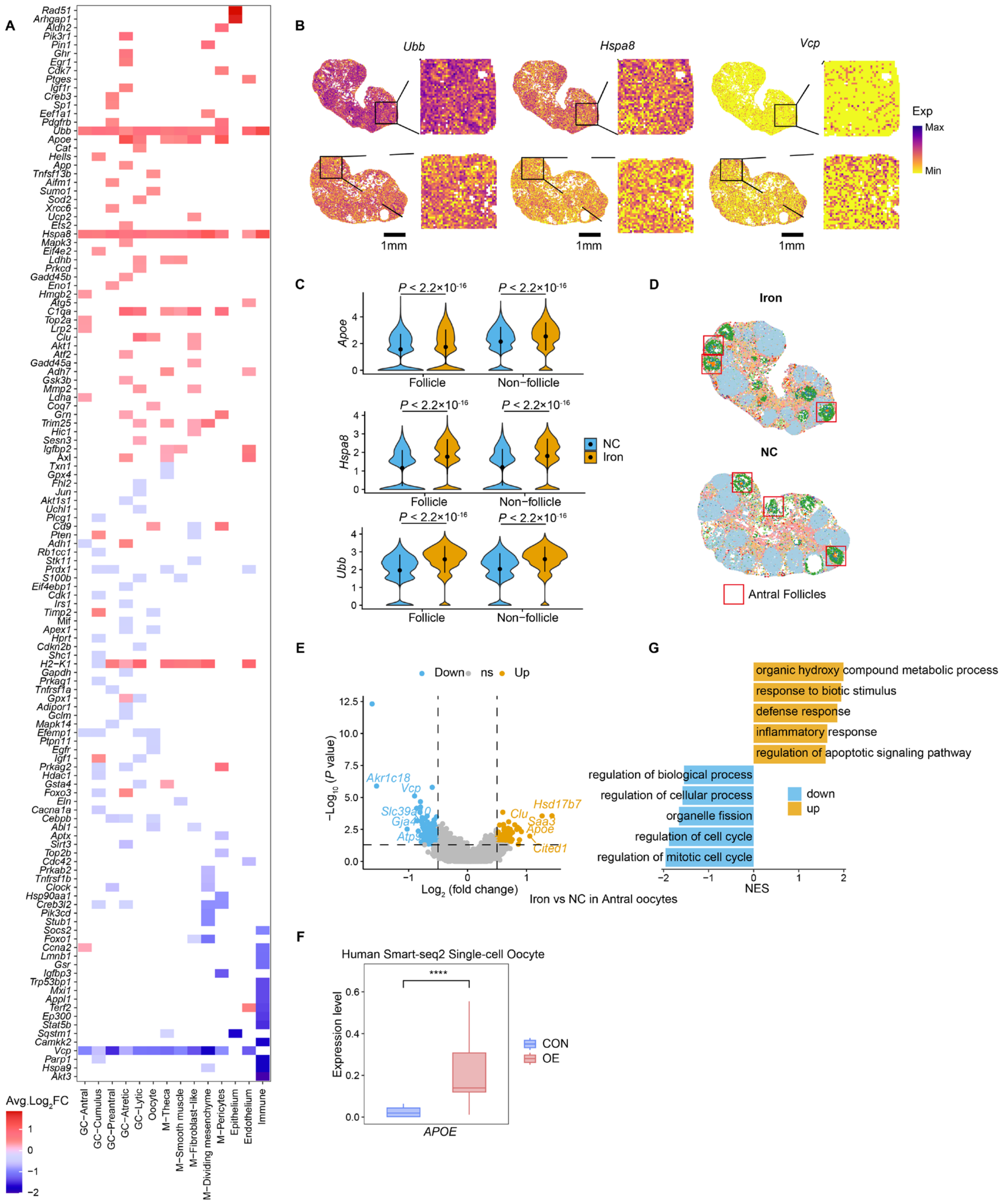

Figure S9. Expression of aging-related genes and transcriptional changes in oocytes under ovarian iron overload, related to Figure 7.

(A) Heatmap displaying the overlap of upregulated and downregulated DEGs across various cell types with genes from the aging atlas database.

(B) Spatial visualization of the expression levels of *Ubb*, *Hspa8*, and *Vcp* genes. Scale bars, 1mm.

(C) Expression levels of *Ubb*, *Hspa8*, and *Vcp* in follicular and non-follicular regions across two groups.

(D) The oocyte transcriptome from antral follicles highlighted in red boxes, as used in (**E**) and (**G**). Each group, n=3.

(E) Volcano plot showing DEGs in antral follicle oocytes between the Iron and NC groups (|Log_2(Fold change)| > 0.5 and P < 0.05).
(F) Comparison of *APOE* gene expression levels in oocytes from OEI patients and control patients, analyzed using Smart-seq2 single-cell sequencing. P values in (**C**, **F**) were calculated using the two-sided Wilcoxon rank-sum test. ****P < 0.0001.
(G) GSEA of GO biological processes for genes ranked by Log_2(Fold change) between oocytes from the Iron and NC groups.

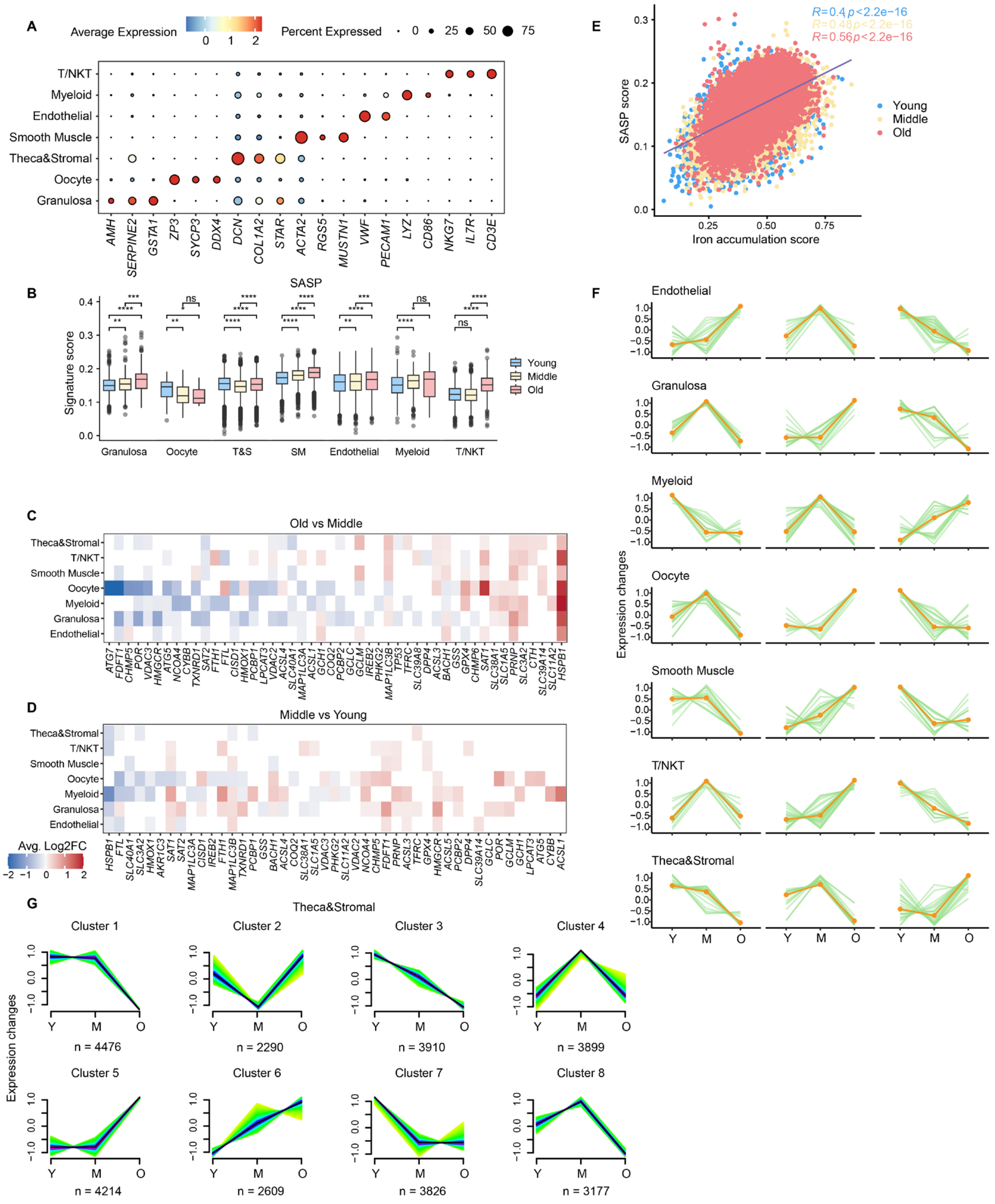

Figure S10. Single-cell atlas of human aging ovaries and dynamics regulation of iron homeostasis, related to Figure 8.

(A) Dot plot annotating the expression levels and percentages of representative markers across seven major cell types in human ovarian tissue.

(B) Gene set scoring analysis of SASP features across different age groups. Two-sided Wilcoxon rank-sum test. *****P* < 0.0001, ****P* < 0.001, ***P* < 0.01, **P* < 0.05, ns, no significance.

(C-D) Heatmaps showing ferroptosis-related DEGs between Old and Middle groups (**C**), and Middle and Young groups (**D**).

(E) Pearson correlation between iron accumulation scores and SASP scores across different groups, with colors representing distinct groups.

(F) Line plot of standardized iron homeostasis gene expression dynamics with age across

different cell types, identified through fuzzy clustering; green lines represent the expression dynamics of individual iron homeostasis genes.

(G) Line plot of standardized gene expression dynamics with age in theca & stromal cells, identified through fuzzy clustering; green lines represent the expression dynamics of individual genes. *n* denotes the number of genes clustered in each group.
