# Supplementary material for: Integrative Single‐Cell Analysis Reveals Iron Overload‐Induced Senescence and Metabolic Reprogramming in Ovarian Endometriosis‐Associated Infertility
Source: Adv Sci (Weinh). 2025 Jul 22;12(29):e17528. doi: 10.1002/advs.202417528 (PMC12362736; doi:10.1002/advs.202417528)
Supplement: Supplementary file 2 — Supporting Tables [file ADVS-12-e17528-s001.pdf]

ADVANCED SCIENCE
Open Access

Supporting Information

for *Adv. Sci.*, DOI 10.1002/advs.202417528

Integrative Single-Cell Analysis Reveals Iron Overload-Induced Senescence and Metabolic Reprogramming in Ovarian Endometriosis-Associated Infertility

Yangshuo Li, Wei Zhou, Jie Ding, Di Song, Wen Cheng, Jin Yu, Shuai Sun, Shanshan Mei, Xiaolan Liang, Qianqian Zhao, Yanping Kuang, Mingqing Li, Zhexin Ni*, Chaoqin Yu* and Yue Gao**

Supplementary table 1 Baseline characteristics in IVF/ICSI population.

	Male factor (n = 308)	OE factor (n = 308)	Tubal factor (n = 308)	*P* value[a]	*P* value[b]
**Age, years**					
Female	31.75 (3.21)	31.73 (3.47)	31.07 (4.02)	0.94	0.030
Male	33.20 (4.18)	33.30 (4.52)	32.75 (5.10)	0.77	0.15
**Female BMI, kg/m²**	21.10 (1.45)	20.84 (2.99)	20.59 (2.11)	0.17	0.24
**Parity**				0.77	0.77
0	301 (97.7%)	303 (98.4%)	301 (97.7%)		
>0	7 (2.3%)	5 (1.60%)	7 (2.30%)		
**Duration of infertility, years**	2.92 (2.59)	2.98 (2.33)	2.68 (2.74)	0.77	0.14
**Number of previous IVF or ICSI cycles**				0.97	0.97
0	196 (63.6%)	199 (64.6%)	202 (65.6%)		
1	83 (26.9%)	80 (26.0%)	77 (25.0%)		
2	29 (9.4%)	29 (9.4%)	29 (9.4%)		
**Ultrasonographic examination**					
Antral follicle count	8.73 (7.44)	4.72 (5.60)	7.33 (7.33)	< 0.001	< 0.001
Endometrial thickness, mm	11.15 (2.36)	11.02 (2.49)	11.03 (2.45)	0.51	0.97
**Basal laboratory testing (female partner)**					
Basal FSH, IU/L	5.70 (2.64)	7.36 (5.64)	5.63 (2.17)	< 0.001	< 0.001
Basal LH, IU/L	5.81 (6.24)	6.52 (7.74)	6.36 (7.20)	0.21	0.79
Basal E_2, pmol/L	62.79 (68.57)	78.37 (78.17)	74.42 (80.13)	0.0087	0.54
**FET endometrial preparation**				0.26	1
Modified natural cycle	92 (30.1%)	106 (34.4%)	107 (34.7%)		
Artificial cycle	216 (70.1%)	202 (65.6%)	201 (65.3%)		

OE, ovarian endometriosis; BMI, body mass index; FSH, follicle-stimulating hormone; LH, luteinizing hormone; E_2, oestradiol; FET, frozen-thawed embryo transfer.

Data are presented as mean (SD) for continuous variables and n (%) for categorical variables.

All *P* values were assessed with the use of Student' t tests or chi-square test.

[a] OE factor vs. Male factor; [b] OE factor vs. Tubal factor.

Supplementary table 2 Oocyte retrieve and embryo transfer index.

	Male factor (n = 308)	OE factor (n = 308)	Tubal factor (n = 308)	*P* value[a]	*P* value[b]
**Follicle count**	4.76 (5.68)	2.79 (3.83)	4.47 (5.68)	<0.001	<0.001
> 10mm Follicle count	1.37 (1.55)	1.07 (1.07)	1.41 (1.59)	0.0055	0.0022
> 14mm Follicle count	1.06 (1.05)	0.81 (0.78)	0.99 (0.90)	<0.001	0.0086
**Number of follicles punctured**	17.74 (11.00)	11.12 (9.51)	16.63 (11.32)	<0.001	<0.001
**Number of oocytes retrieved**	12.91 (8.09)	7.92 (5.67)	12.32 (7.09)	<0.001	<0.001
**Rate of oocytes retrieved**	0.75 (0.22)	0.70 (0.24)	0.71 (0.23)	0.0066	0.43
**Number of metaphase II oocytes**	10.70 (6.62)	6.63 (4.19)	10.37 (5.94)	<0.001	<0.001
**Fertilization method**				<0.001	<0.001
IVF	12 (3.9%)	240 (77.9%)	265 (86.0%)		
ICSI	275 (89.3%)	50 (16.2%)	34 (11.0%)		
IVF + ICSI	21 (6.8%)	18 (5.8%)	9 (2.9%)		
**Number of cleavage zygote**	8.82 (5.52)	5.77 (3.64)	8.90 (5.26)	<0.001	<0.001
**Number of normal zygotes**	8.66 (5.36)	5.66 (3.55)	8.78 (5.16)	<0.001	<0.001
**Number of high-quality embryos**	4.43 (3.25)	2.97 (2.09)	4.74 (3.56)	<0.001	<0.001
**Number of available blastocysts**	0.88 (1.35)	0.61 (1.12)	1.11 (1.57)	0.0060	<0.001
**Number of embryos transferred group**				0.47	0.0028
1	55 (17.9%)	63 (20.5%)	35 (11.4%)		
>1	253 (82.1%)	245 (79.5%)	273 (88.6%)		
**Stage of embryos transferred**				0.41	0.82
Cleavage-stage	271 (82.0%)	263 (85.4%)	266 (86.4%)		
Blastocyst-stage	37 (12.0%)	45 (14.6%)	42 (13.6%)		

Data are presented as mean (SD) for continuous variables and n (%) for categorical variables.
All *P* values were assessed with the use of Student' t tests or chi-square test.
[a] OE factor vs. Male factor; [b] OE factor vs. Tubal factor.

Supplementary table 3 IVF/ICSI Outcomes.

	Male factor (n = 308)	OE factor (n = 308)	Tubal factor (n = 308)	*P* value[a]	*P* value[b]
**Number of live births**	130 (42.2%)	122 (39.6%)	145 (47.1%)	0.57	0.074
**Clinical pregnancy**	130 (42.2%)	123 (39.9%)	145 (47.1%)	0.62	0.088
**Twin pregnancy**	30 (9.7%)	29 (9.4%)	37 (12.0%)	1	0.36
**Gestational age, week**					
Large for gestational age	7 (5.4%)	2 (1.6%)	4 (2.8%)	0.17	0.69
Small for gestational age	16 (12.3%)	21 (17.1%)	23 (15.9%)	0.29	0.87
**Birthweight, g**	3061.79 (580.20)	3013.83 (597.07)	2995.31 (681.33)	0.47	0.79
Singleton, g	3302.60 (562.58)	3305.74 (496.15)	3432.61 (382.02)	0.98	0.046
Twin, g	2609.42 (472.14)	2545.78 (422.93)	2357.08 (491.16)	0.44	0.019
Low birthweight (<2500 g)	20 (12.6%)	26 (17.2%)	42 (23.1%)	0.27	0.22
High birthweight (>4000 g)	5 (3.1%)	5 (3.3%)	6 (3.3%)	1	1
**Congenital anomaly**	2 (1.26%)	8 (5.30%)	1 (0.55%)	0.044	0.0079
**Neonatal anomaly**	2 (1.26%)	5 (3.31%)	2 (1.10%)	0.53	0.060

Data are presented as mean (SD) for continuous variables and n (%) for categorical variables.
All *P* values were assessed with the use of Student' t tests or chi-square test.
[a] OE factor vs. Male factor; [b] OE factor vs. Tubal factor.
